# Injectable and viscoelastic click alginate hydrogels for spatio-temporal T cell administration in vivo

**DOI:** 10.1016/j.mtbio.2026.103128

**Published:** 2026-04-24

**Authors:** Jone Berasain, Sara Manzano, Oihane Mitxelena-Iribarren, Unai Heras, Ainhoa Romo-Valera, Peio Azcoaga, Leire Iturriaga, Leire Egia-Mendikute, Asís Palazón, Mercedes Fernández, María M. Caffarel, Robert Aguirresarobe, Amaia Cipitria

**Affiliations:** a Group of Bioengineering in Regeneration and Cancer, Biogipuzkoa Health Research Institute, San Sebastian, Spain; b Department of Advanced Polymers and Materials: Physics, Chemistry and Technology, Faculty of Chemistry, University of the Basque Country EHU, San Sebastian, Spain; c Group of Breast Cancer, Biogipuzkoa Health Research Institute, San Sebastian, Spain; d Department of Cell Biology and Histology, Faculty of Medicine and Nursing, University of the Basque Country EHU, Leioa, Spain; e Cancer Immunology and Immunotherapy Laboratory, CIC bioGUNE-BRTA, Derio, Spain; f IKERBASQUE, Basque Foundation for Science, Bizkaia, Spain; g POLYMAT, University of the Basque Country EHU, San Sebastian, Spain

**Keywords:** Injectable hydrogel, Viscoelasticity, T cell delivery, Chick chorioallantoic membrane (CAM) model, *In vivo* mouse model

## Abstract

T cell therapies have shown limited success in solid tumors, mainly due to the difficulty of T cells to penetrate the tumor tissue. Here, we develop injectable and viscoelastic click alginate hydrogels for local and sustained delivery of T cells, with the goal to improve T cell administration, viability, proliferation, and persistence *in vivo*. Oxidized alginate (Alg), functionalized with norbornene and tetrazine for inverse electron demand Diels-Alder covalent click crosslinking, at varying low (1% Alg) and high (2% Alg) alginate concentration were used. 1% Alg hydrogels showed better injectability in a fully crosslinked state, characterized by lower stiffness, larger mesh size, viscoelastic behavior, lower injection forces and higher cell viability upon injection. *In vitro* experiments demonstrated that 1% Alg supported T cell viability and proliferation, and promoted sustained release for 10 days. Using an *in vivo* chick chorioallantoic membrane (CAM) model, hydrogel-based T cell administration exhibited better local delivery, proliferation and persistence over time compared to bolus injection, with 1% Alg showing enhanced T cell release compared to 2% Alg. Further, in a murine model with a local injection in the mammary gland, 1% Alg showed enhanced T cell persistence within the mammary gland and high tissue integration. In conclusion, we engineered injectable, viscoelastic click alginate hydrogels that support T cell administration, local injection, viability, proliferation and persistence *in vivo,* opening future opportunities for spatio-temporal control of T cell immunotherapies.

## Highlights

• Tuning alginate concentration and crosslinking density, we can control the injectability and viscoelasticity of click-crosslinked gels.
• Lower concentration enhances viscous relaxation and promotes higher T cell viability, proliferation and sustained release *in vitro.*
• Injectable click crosslinked alginate hydrogels enable T cell transplantation and persistence *in vivo.*
• Hydrogel-based T cell administration showed better local delivery and persistence over time compared to bolus injection in an *in vivo* CAM model.
• Lower alginate concentration showed enhanced T cell persistence and high tissue integration within the mammary gland in an *in vivo* mouse model.

## Graphical abstract

**Figure:**
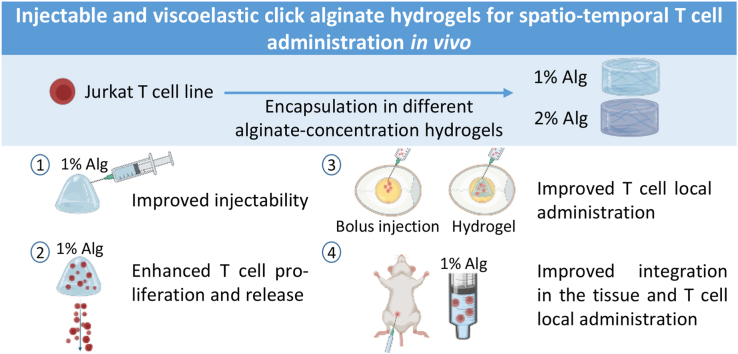

## 1 Introduction

While T cell therapies effectively eliminate circulating tumor cells in hematological malignancies [[1], [2], [3]], their efficacy in solid tumors remains limited by physical barriers, metabolic stress and immunosuppressive cells and cytokines, which impair T cell infiltration, survival and function [[4], [5], [6], [7]]. Consequently, high doses are administered systemically, resulting in increased toxicity [8]. Alternative strategies are needed to enhance local T cell administration, viability, proliferation and persistence *in vivo*, in order to prolong the therapeutic effects [2,9,10]. The spatio-temporal control of T cell administration could increase the continuous contact between T cells and tumor cells, improving the effectiveness of the therapy and limiting the toxicity associated with systemic administration of high doses and consequent high level of released cytokines [[11], [12], [13]].

Injectable hydrogels have demonstrated potential and appropriate characteristics for T cell local administration, including their ability to retain injected cells over time, capacity to support cellular functions, and possibility of being implanted using minimally invasive techniques [14,15]. However, some of the materials described so far are synthetic, such as Grosskopf and Apple's polymer-nanoparticle hydrogels or Lizana-Vasquez and Torres-Lugo's thermoresponsive polymers [16,17]. Clinical translation of these synthetic materials is further complicated by the unpredictable metabolism and degradation, as well as the potential impact of their degradation products on the body [2,18]. Other described injectable hydrogels of natural origin, such as gelatin methacrylate (GelMA), require the presence of a photoinitiator for crosslinking [19]. This also introduces additional challenges for clinical application, as photoinitiators and their derivatives can be cytotoxic, the use of UV or visible light for *in situ* polymerization can damage cells and raises safety issues, as well as inconsistencies with reproducibility in a clinical setting [2,20,21].

The crosslinking mechanism of hydrogels, either non-covalent (physically crosslinked) or covalent (chemically crosslinked), plays a crucial role in determining their physical, mechanical, chemical and biological properties that directly affect hydrogels' suitability for injection and use in cell therapy [22]. Non-covalently crosslinked hydrogels rely on reversible bonds that respond to environmental cues [9,23,24], conferring viscoelasticity and stimuli-responsive gelation, enabling injectability [24,25]. Some advantages they present are the rapid structure recovery after injection, their ability to gel without toxic crosslinkers and improved mimicry of native extracellular matrices (ECM) through energy dissipation under mechanical stress [[25], [26], [27]]. However, they often present instability *in vivo* leading to issues like uncertainty to form gels, rapid and uncontrolled degradation, and high burst release [[27], [28], [29]].

In contrast, covalently crosslinked hydrogels consist of stable bonds, which provide mainly elastic properties and long-term durability, making them attractive for more sustained performance [30]. However, the crosslinking process sometimes involves toxic initiators or harsh reaction conditions, which can compromise biocompatibility [31]. To address this, recent strategies focus on spontaneous covalent crosslinking reactions such as Diels-Alder, inverse electron demand Diels-Alder (IEDDA) and Schiff-base reactions, which also have demonstrated self-healing behavior permitting injection and high biocompatibility [27,[32], [33], [34]]. Despite this, the degradation of covalently crosslinked materials remains a limitation for medical translation. Strategies to address this include the incorporation of matrix metalloproteinase-sensitive crosslinkers, the use of methacrylated biodegradable polymers, or combining highly degradable biomaterials such as gelatin with IEDDA click chemistry [[35], [36], [37]]. Finally, another strategy to improve the degradation of natural biomaterials has been their modification by oxidation, resulting in matrices susceptible to hydrolysis in combination with covalent crosslinking; however, their suitability for injection has not yet been described [38].

To better mimic native tissues, the biomaterials field has advanced by incorporating viscoelasticity as a fundamental property of hydrogels. Unlike purely elastic matrices, which mechanically confine encapsulated cells, viscoelastic materials exhibit time-dependent stress relaxation and deformation [[39], [40], [41]]. This dynamic behavior has been shown to modulate essential cellular processes, including spreading, division, migration and differentiation [39,40,[42], [43], [44], [45], [46]]. Specifically, viscoelasticity is crucial for T cell therapy, as it has been found to directly regulate the transcriptional landscape and activation state of T cells [[47], [48], [49]].

In this context, we developed a covalently crosslinked, injectable, viscoelastic and degradable click alginate hydrogel based on oxidized and functionalized alginate. This hydrogel is naturally derived and demonstrates spontaneous crosslinking, without the need for any external agents such as UV light. Additionally, this hydrogel can be injected in a fully crosslinked state and support T cell expansion and spatio-temporal delivery. First, alginate was oxidized and functionalized with norbornene (N) and tetrazine (T) to enable hydrolytic degradation and covalent IEDDA click crosslinking. In order to potentiate injectability and facilitate T cell proliferation and release, alginate concentration and crosslinking density were tuned. 2% w/v alginate serves as our baseline, supported by extensive characterization of parameters like the N:T ratio [38,[50], [51], [52]]. Here, we evaluated 1% w/v alginate (N:T 1.8) to develop softer, viscoelastic click hydrogels, with increased mesh size and improved injectability in a fully crosslinked state for localized T cell delivery. Rheological measurements and injectability analysis exhibited lower stiffness and viscosity, and a suitable injection force for the lowest alginate concentration. The supportive microenvironment of these hydrogels was demonstrated by the encapsulation and administration of T cells, evaluating cell viability, proliferation and sustained release kinetics. Using an *in vivo* chick chorioallantoic membrane (CAM) model and a mouse model, bioluminescence tracking and immunofluorescence imaging confirmed better T cell administration, proliferation and persistence over time using hydrogels for T cell delivery, compared to bolus injection in media. Finally, histological analysis also confirmed the biocompatibility and degradability of these materials, as tissue infiltration through the hydrogel occurred.

In summary, injectable and viscoelastic click alginate hydrogels exhibited appropriate injectable properties due to their low stiffness, viscoelasticity and reduced injection force, which combined with their degradability provided a suitable environment for T cell local injection, persistence and sustained release *in vivo.* Together with their simplicity in the synthesis and application, as pre-crosslinked T cell-laden gels remain injectable, these hydrogels open future opportunities for spatio-temporal control of T cell immunotherapies.

## 2 Materials and methods

### 2.1 Materials

Materials used for alginate synthesis, hydrogel fabrication and rheological characterization were: High guluronic acid low molecular weight sodium alginate (LMW; M_W_ 75 kDa Pronova UP VLVG; NovaMatrix, #42000501), sodium periodate (99.8%; Sigma-Aldrich, #311448), dialysis membranes (Spectra/Por 6, MWCO 3.5 kDa; Spectrum, #11495869), ammonia borane (97%; Sigma-Aldrich, #12352116), 2-N-morpholino-ethanesulfonic acid (MES; Sigma-Aldrich, #E7750), N-(3-Dimethylaminopropyl)-N′-ethylcarbodiimide hydrochloride (EDC; Sigma-Aldrich, #E6383), N-hydroxysuccinimide (NHS; Sigma-Aldrich, #130672), 5-norbornene-2-methylamine (N, TCI Chemicals, #N0907), 3-(p-benzylamino)-1,2,4,5-tetrazine (T; Conju-probe, #C6021), hydroxylamine (Sigma-Aldrich, #11881405), activated charcoal (Sigma-Aldrich, #C9157), deuterium oxide (99.95%; MagniSlov, #1034280009), Dulbecco's Phosphate-Buffered Saline (PBS, without Ca^2+^, Mg^2+^, and phenol red; Gibco™, #14190144), mineral oil (N350 Viscosity Oil Standard; PSL Rheotek, #2700-V12), siliconizing reagent (Sigmacote; Sigma-Aldrich, #SL2), sterile cell strainers (Fisherbrand™, #11587522), and Kimwipe tissue (Kimberly-Clark, #7552).

Materials used for cell culture, *in vivo* experiments and histology were: 1 mL syringe (Ico plus, #N15663), 25G 5/8" needle (BD Microlance™, #300600), L-Glutamine (Gibco™, #25030081), Pen Strep (Gibco™, #15140122), pCDH-EF1-Luc2-P2A-tdTomato plasmid (Luciferase/tdTomato plasmid; Addgene, #72486), RPMI-1640 (Gibco™, #11879020), DMEM (Gibco™, #11995065), pMDLg-pRRE plasmid (Addgene, #12251), pRSV-REV plasmid (Addgene, #12253), pMD2.G-VSVG plasmid (Addgene, #12259), Turbofect transfection reagent (Thermo Fisher Scientific, #R0531), Calcein AM (Biomol Bioquest, #ABD-22002), ethidium homodimer-1 (Sigma-Aldrich, #E1903), bovine serum albumin (BSA, Sigma-Aldrich, #A7906), formalin (PFA, Sigma-Aldrich #HT501128), Triton X-100 (Sigma-Aldrich, #HFH10), Phalloidin 405 Conjugate (PHA; CruzFluor™, #SC363790), 4′,6-diamidino-2-phenylindole (DAPI; Sigma-Aldrich, #MBD0015), 24-well transwell (Corning, #353097), IVISbrite™ D-Luciferin potassium salt (D-luciferin, Revvity, #122799), Tissue-Tek® optimal cutting temperature Compound (OCT; Sakura, #4583), Harris' hematoxylin (H; Sigma-Aldrich, #1092530500), Eosin Y solution (E; Sigma-Aldrich, #318906), Anti-CD3 epsilon antibody (Abcam, #52959), Goat normal serum (Invitrogen™, #10189722), Anti-mouse Alexa Fluor® 647 Conjugate (Cell Signaling Technology, #4410), and ProLong Gold antifade mountant (Invitrogen™, #P36930).

### 2.2 Modified alginate synthesis

#### 2.2.1 Oxidation of alginate

As previously described [38], hydroxyl groups of 5% of the monomers of LMW (<75 kDa) and high-guluronic acid sodium alginate were oxidized by dissolving at 1% w/v in deionized water (diH_2_O) and reacting with sodium periodate (NaIO_4_) at a molar equivalent relative to the number of monomers to be oxidized. The reaction mixture was stirred at 250 rpm in the dark at room temperature (RT) overnight. The solution was purified by dialysis in diH_2_O with 3-4 water changes per day for 3 days. Finally, the purified oxidized alginate was lyophilized.

#### 2.2.2 Reduction of oxidized alginate

The partially oxidized LMW alginate was then reduced using ammonia borane. The polymer was dissolved at 1% w/v in diH_2_O and reacted with ammonia borane (BH_3_NH_3_) at a molar equivalent to the number of monomer units to be reduced, by stirring at 250 rpm for 16 h at RT in the dark. The resulting solution was dialyzed and lyophilized as before.

#### 2.2.3 Alginate functionalization by carbodiimide chemistry

Oxidized and reduced LMW alginate was dissolved at 1% w/v in MES buffer (0.1 M, pH 6.5). Then, EDC and NHS were added drop-wise to the alginate solution at 5000 molar equivalents each. EDC and NHS were allowed to react with alginate for 5 min at RT with gentle stirring.

To functionalize the oxidized LMW alginate backbone with the functional groups, N was introduced at a theoretical degree of substitution (DS_theo_) of 500 molecules per alginate chain, while T was added at a DS_theo_ of 170. Both reactions proceeded at RT for 18 h with constant stirring at 700 rpm. Then, hydroxylamine was used for quenching. The resulting solutions were first purified by dialysis as described previously and then treated with activated charcoal for 30 min. Finally, the solutions were sterile-filtered and lyophilized.

#### 2.2.4 Proton nuclear magnetic resonance (^1^H NMR) characterization of modified alginates

To confirm the oxidation and reduction of the LMW alginate chain and determine the actual degree of substitution (DS_actual_) of N and T, ^1^H NMR analyses were performed. For this purpose, samples were dissolved in deuterium oxide at 1.5% w/v concentration, and 64 scans were acquired for each measurement using an Ultra Shield Plus 500 MHz spectrometer (Bruker). Then, the spectra were analyzed using MestreNova software (v12.03).

### 2.3 Hydrogel fabrication

Lyophilized N-modified (N-mod) and T-modified (T-mod) alginate polymers were dissolved in PBS separately. The dissolved N-mod and T-mod were then mixed at a norbornene:tetrazine (N:T) molar ratio of 1.8 and final alginate concentrations of 1% and 2% (w/v). From this point onward, the hydrogels will be referred to as “1% Alg” and “2% Alg”.

To fabricate preformed non-injected cylindrical hydrogels, the mixed solution was pipetted into a silicone mold (5 or 8 mm diameter and 2 mm height) placed on a glass slide and covered with a siliconized glass slide. The mold was then placed in an incubator at 37 °C and allowed to gel for 2.5 h. Then, the mold was peeled off and the non-injected cylindrical hydrogels were obtained.

To generate the injectable hydrogels, N-mod and T-mod were carefully mixed and the solution was pipetted into a syringe. After 2.5 h, a 25G 5/8" needle was assembled so the hydrogels could be injected.

### 2.4 Hydrogel characterization

#### 2.4.1 Degradability assays

The degradability of the hydrogels was evaluated by monitoring wet and dry weight changes during incubation in RPMI medium at 37 °C under a CO_2_ atmosphere. Samples were placed individually in pre-weighed 40 μm pore cell strainers and weighed together with them at each time point. Wet weight was recorded on days 1, 3, 7, 10, 21, and 28 after removing excess liquid with Kimwipe tissue, while dry weight was determined on days 0, 3, 7, and 10 following lyophilization. For calculations, the weight of each strainer was subtracted from the total weight. For the analysis, n = 3 samples per group were used.

#### 2.4.2 Rheological properties

The rheological properties of the hydrogel precursor solutions and crosslinked hydrogels were characterized using an ARES-G2 rheometer (TA Instruments) with a 25 mm cone plate geometry with a 0.04 rad cone angle. Also, mineral oil was added in the periphery to prevent hydrogel dehydration. For the analysis, N-mod and T-mod solutions were mixed and immediately pipetted onto the bottom plate of the rheometer. Then, flow behavior, crosslinking kinetics, and elastic and viscoelastic properties were characterized. The viscosity of non-crosslinked hydrogels was measured with a shear rate ranging from 1 to 10 s^−1^. Next, crosslinking kinetics were analyzed by time sweep experiments at a constant frequency of 1 Hz. Once gelation of the hydrogels was complete, a frequency sweep was conducted from 0.1 to 1 Hz to determine the storage (G') and loss (G") moduli. Finally, a stress relaxation test was conducted by applying a 50% strain for 5 h. Time sweep, frequency sweep and stress relaxation experiments were performed sequentially on the same hydrogel. All measurements were carried out at 37 °C, under linear viscoelastic conditions confirmed by strain sweep tests.

Data analysis was performed using the TA Instruments Trios software (v5.1.1). The crossover points of G' and G" in the time sweep were used to determine the gelation point. To obtain the elastic modulus, first the complex shear modulus (G∗) was derived from G' and G" in the plateau using Rubber's elasticity theory$\left|G^{*}\right|=\sqrt{{\left(G^{\prime }\right)}^{2}+{\left(G^{\prime \prime }\right)}^{2}}$. Then, the elastic modulus (E) was calculated using the average of G∗ values and the approximation of Poisson's ratio (ϑ) equal to 0.5, $E=2G^{*}\left(1+\vartheta \right)$. The mesh size was estimated by using the formula: $\xi =\sqrt[{3}]{6RT/G^{\prime }\pi N_{av}}$ as previously described [53]. Finally, the stress relaxation half time (τ_1/2_) was determined as the characteristic time required for the material to relax 50% its initial modulus in stress relaxation tests. For the analysis, n = 3 samples per group were used.

#### 2.4.3 Mechanical properties and injectability test

Injection force measurements were conducted using a Texture Analyzer TA.XTPlus (Stable Micro Systems) equipped with a 50 N load cell. N-mod and T-mod were mixed and immediately loaded into 1 mL syringe with 25G 5/8" needle. To assess the effect of crosslinking time on injectability and simulate injection, the plunger was compressed at 0, 15, 60, 120 and 180 min, at a constant rate of 1 mm/s and at RT. The injection force was calculated as the average force within the plateau region of the force-displacement curve during extrusion. For the analysis, n = 3-4 samples per group were used.

#### 2.4.4 3D porosity analysis by microcomputed tomography (μCT)

For porosity analysis, both 1% Alg and 2% Alg were snap-frozen and lyophilized overnight prior to μCT imaging. Samples were imaged using the SkyScan 1172 high-resolution μCT system (Bruker), equipped with a Hamamatsu Photonics 100/250 X-ray source and a Hamamatsu C9300 11-megapixel camera. Hydrogels were scanned at a voltage of 48 kV and a current of 167 μA, without the use of an additional beam filter. Images were acquired with a voxel size of 6.85 μm and collected over an angular range of 192.5°, using a rotation step of 0.25° in a counterclockwise direction. Lastly, reconstruction of cross-sectional images was performed using NRecon (NReconServer v1.7.4) in standard mode, followed by 3D volume rendering and analysis using ORS Dragonfly software, version 2024.1 (Comet Technologies Canada Inc.). For the analysis, n = 3 samples per group were used.

### *2.5* Functional evaluation of T cell behavior and release kinetics in injectable hydrogels

#### 2.5.1 T cell lines

Jurkat cell lines were cultured as a model for T lymphocytes. For cell tracking, a Luciferase/tdTomato reporting Jurkat cell line was generated (Jurkat-TOM). Briefly, for lentiviral production, HEK-293T cells were co-transfected with standard packaging and envelope plasmids, along with a Luciferase/tdTomato expression vector kindly provided by the Cancer Stem Cells and Fibroinflammatory Microenvironment group at the Instituto de Investigaciones Biomédicas “Alberto Sols" (IIBm-CSIC), Madrid. After 24 h, the viral supernatant was collected and filtered through a 0.45 μm filter before being used to transduce Jurkat cells. 48 h later, verification of successful transduction and sorting of the Jurkat-TOM cell line were performed using the H800S Cell Sorter (SONY).

The Jurkat and Jurkat-TOM cell lines were cultured in RPMI-1640 medium supplemented with 10% v/v fetal bovine serum, 1% L-Glutamine and 1% Pen Strep. For HEK-293T cells, DMEM was used with the same supplementation. Cells were incubated in a 5% CO_2_ environment at 37 °C. Jurkat cells were passaged when a cell density of 1·10^6^ cells/mL was reached and HEK-293T cells when confluence of 70% was reached. For encapsulation in hydrogels, a density of 1·10^6^ cells/mL was chosen, following established protocols for T cell delivery hydrogels [54].

#### 2.5.2 T cell encapsulation and injectability

Next, the injectability of T cell-laden 1% Alg and 2% Alg, and survival of T cells upon injection were evaluated. Jurkat T cells were encapsulated within the hydrogels by adding the cell suspension in RPMI 1640 medium into the N-mod alginate, prior to mixing with T-mod alginate, at a final concentration of 1·10^6^ cells/mL. After mixing, at 0, 15, 60, 120, and 180 min, 50 μL of the hydrogels were injected through a 25G 5/8" needle into a Live/Dead staining solution containing 1:2000 Calcein AM and 1:1000 ethidium homodimer-1. Following 15 min of incubation at RT in the dark, images were taken with Axio Observer 7 inverted epifluorescence microscope (Carl Zeiss Microscopy). A 100 μm z-stack with 15 μm step size was acquired for each hydrogel. The z-stacks were then collapsed to a 2D maximum projection image for quantification using Dragonfly software, version 2022.2. Cell viability was quantified by calculating the percentage of live cells relative to total cells (sum of live and dead cells). For the analysis, n = 3-4 samples per group were used.

#### 2.5.3 T cell viability, proliferation and morphology

In order to evaluate the applicability of 1% Alg and 2% Alg for T cell administration, Jurkat cells (1·10^6^ cells/mL) were encapsulated and cultured for 10 days. On days 1, 3, 7 and 10, cell viability and growth were evaluated by Live/Dead and DAPI/PHA staining.

Hydrogels were placed into Live/Dead solution containing Calcein AM (1:2000) and ethidium homodimer-1 (1:1000) to identify live and dead cells, respectively. After incubating for 15 min at RT in the dark, images were taken using the epifluorescence microscope. To evaluate cell morphology and proliferation, DAPI/PHA staining was used. For this purpose, hydrogels were first fixed in 4% PFA for 30 min at RT. Then, they were washed twice using 3% BSA in PBS. Cell membrane permeabilization was performed using 0.3% Triton X-100 and leaving for 10 min on the rocker at RT. After removing Triton X-100 and washing again with 3% BSA, PHA (1:40) was added and incubated overnight at 4 °C in the dark. After a final wash with 3% BSA, DAPI (1:1000) was added and incubated for 30 min at RT in the dark. Finally, the hydrogels were placed in PBS before imaging under the LSM900 confocal microscope (Carl Zeiss Microscopy).

For Live/Dead and DAPI/PHA staining imaging, 50 μm z-stacks with 15 μm step size were acquired at 2 positions per hydrogel, for n = 3 hydrogels per group. As previously explained, z-stacks were collapsed to 2D maximum projection images for quantification of cell viability as the ratio of live cells to total cells (sum of live and dead cells) counted using Live/Dead staining, imaged with the epifluorescence microscope. Due to the formation of dense cellular clusters after 7 days, the number of viable cells was determined by subtracting the count of dead cells from the total cell population, as identified by DAPI^+^ nuclei in DAPI/PHA staining, imaged using the confocal microscope. Quantification of cell numbers to evaluate viability and proliferation at each time point was carried out using Dragonfly software.

#### 2.5.4 T cell release kinetics

To assess the T cell release kinetics from 1% Alg and 2% Alg over time, Jurkat cells were encapsulated (1·10^6^ cells/mL) and the number of released cells from the hydrogels to the media was quantified until day 10.

First, as described before, Jurkat cells were encapsulated in 1% Alg and 2% Alg by mixing the cells in N-mod and then adding T-mod. After crosslinking for 2.5 h, 50 μL of both 1% Alg and 2% Alg were injected in a transwell with an 8 μm pore size and cultured in 1.5 mL of complete RPMI-1640 medium. On days 1, 3, 7 and 10, transwell inserts were removed from the well, media were collected and centrifuged at 500 rcf for 5 min. Then, each cell pellet was resuspended in 250 μL of PBS with 1 mM EDTA and DAPI (1:1000), and incubated for 15 min in the dark at RT. The number of cells that were released from the gel to the media was quantified using the CytoFLEX flow cytometer (Beckman Coulter). DAPI^+^ cells were considered as dead, and the number of released viable cells was determined by subtracting dead cells from the total cell count. Data were analyzed using CytExpert software (v2.6; Beckman).

In order to visualize the cell release kinetics over time, Jurkat-TOM cells were encapsulated in non-injected cylindrical hydrogels and cultured under the same conditions. Imaging using the epifluorescence microscope was performed on days 1, 3, 7 and 10, focusing on the well bottom to visualize the hydrogels and the released cells. For the analysis, n = 3-4 samples per group were used.

#### *2.5.5* Functional phenotype of encapsulated T cells through cytokine release analysis

Jurkat cells (1·10^6^ cells/mL) were encapsulated in 1% Alg and cultured for 10 days, using 2D-cultured T cells as a functional control. Conditioned media from both encapsulated and 2D control groups were collected on days 1 and 10, snap-frozen, and stored at -80 °C for subsequent analysis.

IL-2 and IFN-ℽ secretion was quantified using the Olink® Target 48 Human Cytokine panel (Olink Proteomics), which utilizes Proximity Extension Assay (PEA) technology. In PEA, pairs of antibodies conjugated to unique DNA oligonucleotides bind to their target proteins in close proximity, enabling hybridization and extension to form a unique DNA reporter sequence. This reporter is subsequently amplified and quantified by real-time qPCR, allowing for highly specific and sensitive multiplexed detection from small sample volumes.

The assay was performed by the COBIOMIC Bioscience platform (Spain) following the manufacturer's instructions. Protein concentrations were reported in absolute units (pg/mL) based on internal calibration standards. Quality control included the exclusion of proteins or samples that failed the manufacturer's detection thresholds. For the analysis, n = 3 samples per group were used.

### 2.6 Spatio-temporal T cell administration with injectable hydrogels using an *in vivo* CAM model

#### 2.6.1 Eggs preparation and hydrogel injection

Eggs fertilized for 1 day were purchased from Granja Santa Isabel (Spain) and incubated vertically for 10 days at 37.5 °C in a 55% humidified atmosphere, with 45-min scheduled rotations, using the Ovation 56 Ex incubator (Brinsea). As previously described [55,56], on day 10, a hole was drilled in the air chamber at the bottom of the egg using a diamond pen. Then, the air chamber was moved up by creating another hole at the site of interest in the upper part, and vacuuming with a pipette filler. A window fragment was opened around the second hole in the upper part to access the CAM.

Jurkat-TOM cells were encapsulated as previously described in 1% Alg and 2% Alg and loaded into a 1 mL syringe. 50 μL of the T cell-laden gels were inoculated by inserting the 25G 5/8" needle 2 mm through the CAM, preferably near a blood vessel. As a negative control, a bolus injection of Jurkat-TOM cells in RPMI medium, maintaining the same density and volume, was performed. Finally, the windows above the CAM were closed with parafilm and the eggs were further incubated up to 7 days, under the same environmental conditions but without the rotation setting.

#### 2.6.2 Eggs opening and IVIS imaging

In order to evaluate the local T cell administration and retention capacity of the hydrogels, Jurkat-TOM cell presence in proximal CAM was measured by tracking luciferase expression for 7 days using an IVIS Lumina XRMS Series III (Perkin Elmer). First, windows were completely opened, and a total of 150 μL of D-luciferin (30 mg/mL) was added on top of the hydrogels and incubated for 10 min in the dark before image acquisition. The signal was quantified as the total photon flux per second (p/s) in the region of interest that covers the proximal CAM, along with signal area. For the analysis, n = 4-6 samples per group were used.

### 2.7 Spatio-temporal T cell administration with injectable hydrogels using an *in vivo* mouse model

#### 2.7.1 Hydrogel injection in mice

A mouse model was also used to investigate *in vivo* local persistence of T cells using 1% Alg and comparing it with a bolus injection. All procedures in this work were performed in accordance with European regulations and were approved by Biogipuzkoa Committee of Animal Experimentation and Gipuzkoa Regional Government (ethics identification code: PRO-AE-SS-341).

Jurkat-TOM cells were encapsulated in 1% Alg and in RPMI medium (1·10^6^ cells/ml). On day 0, female B-NDG mice (Inovit) aged 5-7 weeks were anesthetized with 3-5% isoflurane with 1-2 L/min oxygen, and maintained during the different procedures with 1-3% isoflurane with 0.5-1 L/min oxygen. 100 μL of 1% Alg or medium was injected into the right fourth mammary gland using a 25G 5/8" needle. For the 1% Alg vs. bolus-injected groups, a total of n = 9 mice per group were used and monitored over 7 days.

#### 2.7.2 In vivo IVIS imaging

Luciferase activity was monitored by bioluminescence (BLI) imaging with the IVIS for 7 days. D-luciferin (150 mg/kg in PBS) was injected intraperitoneally, and after 10 min, mice were anesthetized with isoflurane for imaging. The signal was quantified as the total flux (p/s) in the region of interest. The region of interest was defined as a circular region around the injection zone. Longitudinal live animal imaging was performed on days 1, 2, 4 and 7.

#### 2.7.3 Ex vivo IVIS imaging

On day 7, for ex vivo imaging of the injected mammary glands, D-luciferin (150 mg/kg in PBS) was injected intraperitoneally prior to sacrifice. After 15 min, the animal was sacrificed and organs were dissected. The mammary glands, where the injection was performed, were removed, placed on a Petri dish with D-luciferin (1 mg/mL in PBS) and imaged with the IVIS as previously described.

#### 2.7.4 Histological analysis

For histological and immunofluorescence analysis, injected mammary glands from randomly selected 6 mice per group were collected and snap-frozen in OCT. 25 μm-thick cryosections were obtained at -35 °C using a cryostat (CM1950, Leica Microsystems). To evaluate the condition of the mammary gland tissue and the integration of the hydrogel, sections were stained with H&E following standard protocols.

##### 2.7.4.1 T cell detection by immunofluorescence

For T cell detection by immunofluorescence using a CD3 marker, cryosections were first fixed in 4% PFA for 10 min at RT, blocked with 10% normal goat serum in PBS for 1 h at RT, and incubated overnight at 4 °C with primary anti-CD3 antibody (1:250). After washing with PBS, sections were incubated for 1 h with the secondary antibody Alexa Fluor® 647 Conjugate (1:500) and DAPI (1:1000) and mounted with ProLong mountant.

### 2.8 Statistical analysis

All experiments were conducted with n = 3-9 samples per group. Statistical analyses were performed using GraphPad Prism 10 software (GraphPad Software Inc.). First, normality of each distribution was assessed using the Shapiro-Wilk Test. For normally distributed data, statistical comparison was performed using one-way ANOVA and *t*-test comparisons. Bar plots represent mean ± standard deviation in the graphs. Besides, data with non-normal distribution was analyzed using Kruskal-Wallis and Mann-Whitney tests. When comparing paired data, Friedman's test was used. Box plots show the median (line), 1^st^ and 3^rd^ quartiles (box), and min and max values (whiskers) in the graphs. In all comparisons, a p-value below 0.05 was considered statistically significant. In graphical illustrations, ∗ was used to represent statistically significant comparisons between day/minute 0 and other time points within each experimental group (1% Alg, 2% Alg, or media), while # was used to indicate differences between experimental groups at the same time point (1% Alg vs. 2% Alg vs. media) (∗p < 0.05, ∗∗p < 0.01, ∗∗∗p < 0.001).

## 3 Results

### 3.1 Functionalization of degradable alginate enables IEDDA click crosslinking

To render LMW alginate into a degradable polymer, alginate was oxidized. Specifically, 5% of the hydroxyl groups in the alginate backbone were oxidized using sodium periodate, a process that converts uronic acid residues into open-chain structures capable of undergoing hydrolysis, as previously reported [34,38]. Then, the aldehyde groups were reduced using ammonia-borane to avoid reactions with click-reactive fragments. ^1^H NMR analysis was performed to confirm oxidation and reduction reactions (Supplementary Fig. S1A).

The oxidized LMW alginate was subsequently functionalized with N and T groups through carbodiimide chemistry, with a DS_theo_ of 500 and 170, respectively. The efficiency of these modifications was confirmed by ^1^H NMR spectroscopy and DS_actual_ for two different batches of modified alginate is shown in the Supplementary (Fig. S1B, C; Supplementary Table S1). Those values served as references to maintain a N:T ratio of 1.8 for the desired final polymer concentration. Chemical scheme of the crosslinking reaction is represented in Supplementary Fig. S2. In order to tune the mechanical properties, injectability and cell release profiles, hydrogels with a final alginate concentration of 1% and 2% (w/v) were generated.

### 3.2 Viscosity, crosslinking kinetics, stiffness, viscoelastic properties and porosity in click alginate hydrogels can be tuned by concentration

For the rheological characterization of 1% Alg and 2% Alg, viscosity curves, time sweeps, frequency sweeps, and stress relaxation tests were performed to evaluate the viscosity, crosslinking kinetics, stiffness and viscoelastic properties of the hydrogels, respectively. Linear viscoelastic conditions were ensured by strain sweep tests (Supplementary Fig. S3).

**Fig. 1:**
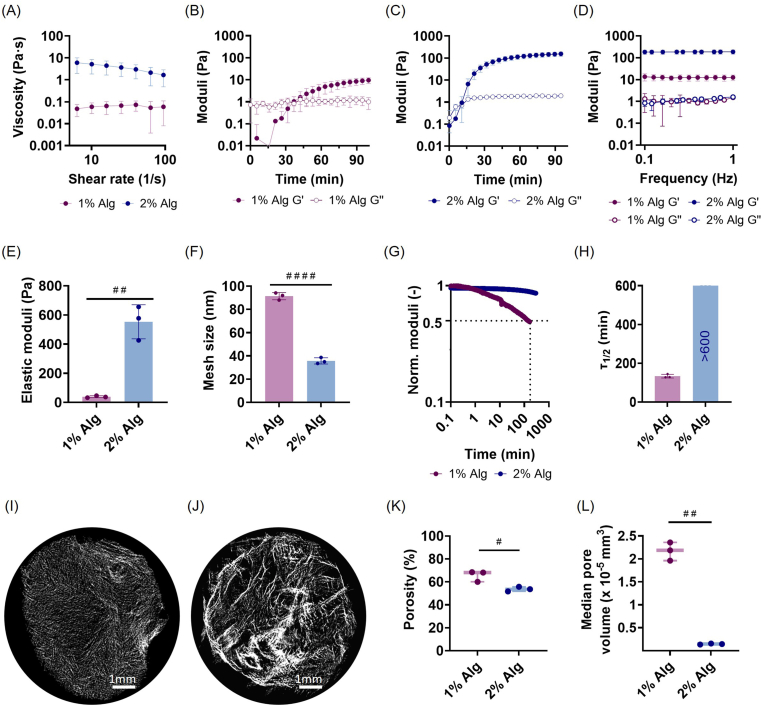
Rheological and structural characterization of 1% Alg in magenta and 2% Alg in blue. Flow curves (A), time sweeps of 1% Alg (B) and 2% Alg (C), frequency sweep (D), elastic modulus (E), mesh size (F), stress relaxation curves (G), stress relaxation time τ_1/2_ (H), μCT images of 1% Alg (I) and 2% Alg (J), porosity (K) and pore volume (L). Bar plots represent mean ± standard deviation, n = 3 per group. Comparisons between 1% Alg and 2% Alg were performed using a t-test. Statistically significant differences are represented with #. (For interpretation of the references to color in this figure legend, the reader is referred to the Web version of this article).

Viscosity curves showed that, prior to crosslinking, both 1% Alg and 2% Alg exhibited very low viscosity (<10 Pa·s) (Fig. 1A), being the viscosity of 1% Alg lower than the one of 2% Alg. While 1% Alg presented a Newtonian behavior, the viscosity decreased in 2% Alg, suggesting a shear-thinning behavior, accompanied by visual signs of gelation due to faster crosslinking.

Time sweep analysis showed the evolution of G' and G" over time (Fig. 1B and C). In the beginning, both 1% Alg and 2% Alg presented higher values of G" than G', indicating the liquid behavior of the materials before crosslinking. The gelation point, defined here as the crossover between G' and G", occurred later in 1% Alg compared to 2% Alg, at around 40 and 15 min, respectively (Fig. 1B and C). Complete crosslinking was confirmed when G' reached a plateau, indicating that hydrogels were fully crosslinked, with 1% Alg exhibiting a G' of 13 Pa, lower than 2% Alg, with 184 Pa (Fig. 1B and C). The subsequent frequency sweep analysis confirmed the hydrogel-like behavior of the materials, as G' and G" were independent of the frequency (Fig. 1D). Additionally, G' and G" values in the frequency sweep were consistent with those observed in the time sweep.

In addition, since the elastic modulus (E) is calculated from G' and G", 1% Alg exhibited significantly lower stiffness (39 ± 7 Pa) compared to 2% Alg (553 ± 117 Pa) (Fig. 1E). Next, the hydrogel mesh size was estimated based on G' values, and results for 1% Alg (91.36 ± 3.11 nm) were significantly higher compared to 2% Alg (35.66 ± 2.67 nm), indicating a more open mesh and higher porosity (Fig. 1F). The stress relaxation test revealed that a reduction of the normalized modulus to 50% occurred in 2 h for 1% Alg, while 2% Alg showed no significant relaxation even after 5 h (Fig. 1G and H). The τ_1/2_ for 1% Alg was 133.4 ± 9.5 min, while 2% Alg did not relax throughout the 5 h test (Fig. 1G and H).

μCT was utilized to quantitatively analyze the physical and architectural properties of the lyophilized hydrogels (Fig. 1I–L, Supplementary Fig. S4). 1% Alg exhibited a significantly higher porosity (65.55 ± 4.80%) compared to 2% Alg (53.70 ± 2.10%) (Fig. 1K). Additionally, the median pore volume was significantly higher for 1% Alg (2.17·10^−5^ ± 2.81·10^−6^ mm^3^) compared to 2% Alg (1.44·10^−6^ ± 9.8·10^−7^ mm^3^) (Fig. 1L). Uniform composition and consistent crosslinking throughout the matrix were demonstrated by the homogeneous distribution of porosity across the scaffold height (Supplementary Fig. S4). Specifically, no statistically significant differences in porosity were found between the top, central and bottom 700 μm sections of the lyophilized hydrogels (Supplementary Fig. S4B). Regarding the pore size distribution through the lyophilized hydrogels, no statistical difference was observed for either of the hydrogels (Supplementary Fig. S4C).

Lastly, the degradability of the hydrogels was confirmed by an observed loss of ≈ 90% of the initial mass in 28 days (Supplementary Fig. S5A). In addition, a significant decrease in the dry weight of 1% Alg was observed after 3 days (Supplementary Fig. S5B).

### 3.3 Optimal injectability is characterized by low extrusion force and high cell viability

#### 3.3.1 Mechanical properties and injectability test of click alginate hydrogels

**Fig. 2:**
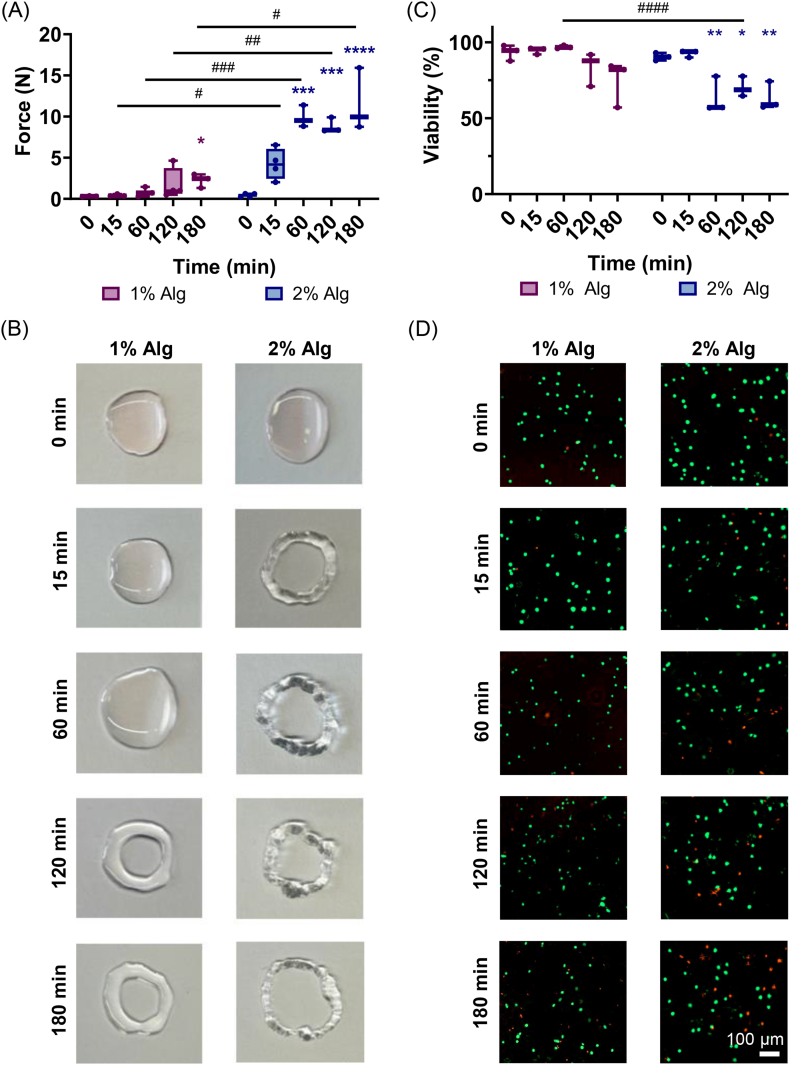
Injectability assays of 1% Alg in magenta and 2% Alg in blue. Injection force analysis (A), hydrogel morphology after injection (B), post-injection T cell viability (C), and Live/Dead staining images (D). Box plots show median, 1^st^ and 3^rd^ quartiles, and min and max values; n = 3–4 per group. Comparisons between 1% Alg and 2% Alg were performed using a Mann-Whitney test. Statistically significant differences are represented with #. Comparisons within each group, with respect to time 0 were performed by Mann-Whitney test in A and Friedman's test in C. Statistically significant differences are represented with ∗. (For interpretation of the references to color in this figure legend, the reader is referred to the Web version of this article).

To mechanically assess the injectability and suitability for injection in a clinical setting, the injection force of 1% Alg and 2% Alg during the gelation process was measured. The injection force was calculated as the average force within the plateau region of the force-displacement curve during extrusion (Supplementary Fig. S6). In the beginning of the gelation process, both 1% Alg and 2% Alg required minimal force to be injected, 0.31 ± 0.04 N and 0.43 ± 0.21 N, respectively (Fig. 2A). After 15 min, a higher injection force was found in 2% Alg, although it was not statistically significant. This increase coincides with the gelation point, evidenced by the crossover of G' and G" observed in the time sweep for 2% Alg. In contrast, 1% Alg maintained nearly the same values (0.43 ± 0.14 N), consistent with the slower gelation kinetics and later gelation point in the time sweep. As more crosslinks were formed, 2% Alg exhibited statistically higher injection forces at 60, 120 and 180 min, with respect to 0 min, reaching values of 9.91 ± 1.33 N, 8.85 ± 0.91 N and 11.55 ± 3.84 N, respectively. However, 1% Alg showed a statistically higher value only when fully crosslinked at 180 min, reaching 2.25 ± 0.84 N. Prior to that, values were not statistically different from 0 min, showing 0.82 ± 0.59 N and 1.79 ± 1.92 N at 60 and 120 min, respectively. Comparing the injection forces between 1% Alg and 2% Alg, statistically higher forces were observed in 2% Alg from 15 min onwards compared to 1% Alg (Fig. 2A).

In terms of appearance and practical injectability, both gels initially exhibited little resistance and were simple to inject while liquid (Fig. 2B). As 2% Alg crosslinked at 15 min, more force was required, and the resistance was considerable. Furthermore, the images demonstrated its rough texture (Fig. 2B). In contrast, regardless of the gelation phase, 1% Alg was easy to inject and had a smooth texture until 180 min (Fig. 2B). These results are consistent with the rheological analysis (Fig. 1), with faster crosslinking kinetics, higher stiffness and elastic nature of 2% Alg resulting in the rough texture, compared to the slower crosslinking kinetics, lower stiffness and viscoelastic nature of 1% Alg resulting in a smoother texture and easier injection (Fig. 2B).

#### 3.3.2 Cell encapsulation and viability upon injection of click alginate hydrogels

After mechanically evaluating injectability, the effect of injection on T cell viability was analyzed in order to evaluate hydrogels' suitability to inject cells. Injecting the hydrogels at 0 min, immediately after mixing N-mod and T-mod, showed a high cell viability in both 1% Alg and 2% Alg, with 93.32 ± 5.18% and 94.51 ± 2.19% of live cells, respectively (Fig. 2C and D). Additionally, no significant decrease in cell viability was found in either 1% Alg or 2% Alg at 15 min. However, from 60 min onwards, a decrease in viability in 2% Alg was found compared to values at 0 min, where viability percentage reached values of 77.74 ± 1.78%, 67.15 ± 2.18% and 63.53 ± 9.31% at 60, 120 and 180 min respectively. In 1% Alg, in contrast, cells showed a high viability across all time points, with values of 96.81 ± 1.05%, 83.48 ± 11.09%, and 74.44 ± 15.11% at 60, 120, and 180 min, respectively (Fig. 2C and D). Moreover, at all time points, 1% Alg showed higher cell viability compared to 2% Alg, although the differences were not statistically significant (Fig. 2C and D).

### 3.4 Click alginate hydrogels enable strong T cell proliferation and high cell release while supporting cell phenotype

#### 3.4.1 T cell viability and proliferation in click alginate hydrogels

In order to evaluate cell viability, proliferation and morphology in 1% Alg and 2% Alg, Jurkat T cells were encapsulated in non-injected cylindrical hydrogels and cultured for 10 days. On days 1, 3, 7, and 10, Live/Dead and DAPI/PHA staining were performed to quantify cell viability, proliferation and morphology.

**Fig. 3:**
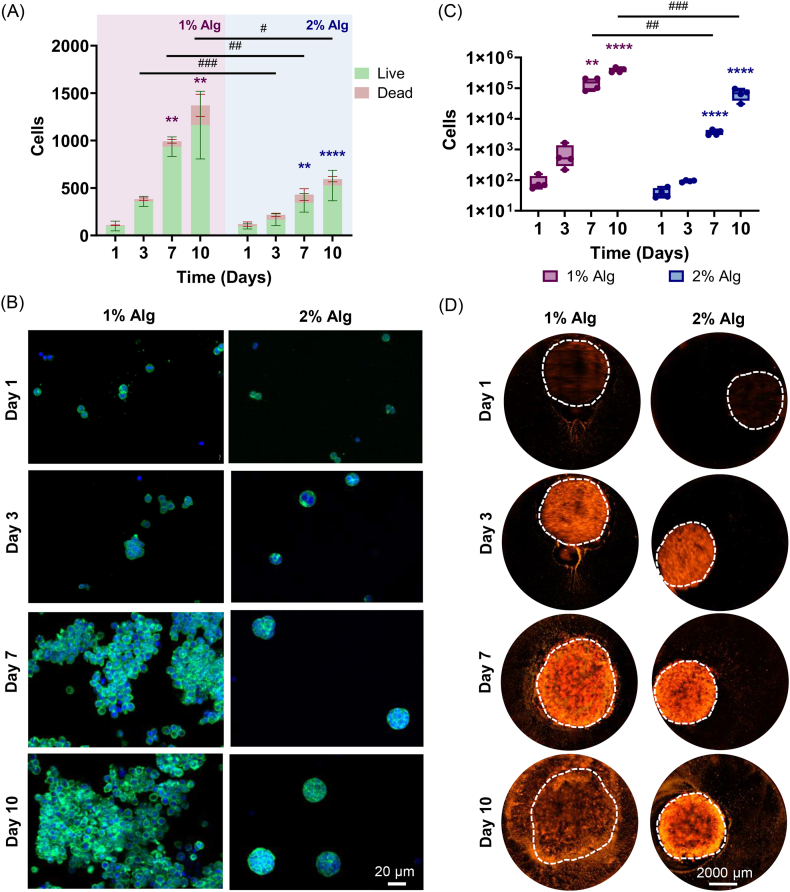
T cell encapsulation in non-injected hydrogels, viability, proliferation and release from hydrogels to media. Total cell number divided into live and dead cells in 1% Alg in magenta box and 2% Alg in blue box (A), DAPI/PHA staining (B), quantification of T cells outside 1% Alg in magenta and 2% Alg in blue (C) and microscopic images of released Jurkat-TOM cells where hydrogels' periphery is marked with a white dashed line (D). For A, bar plots represent mean ± standard deviation, n = 3 per group. For C, box plots show the median, 1^st^ and 3^rd^ quartiles, and min and max values; n = 4 per group. Comparisons between 1% Alg and 2% Alg were performed using a *t*-test in A (comparing number of live cells) and Mann-Whitney test in C. Statistically significant differences between groups are represented with #. Comparisons within each group with respect to time 0 were performed by one-way ANOVA in A and Mann-Whitney test in C. Statistically significant differences are represented with ∗. (For interpretation of the references to color in this figure legend, the reader is referred to the Web version of this article).

Live/Dead staining showed that both 1% Alg and 2% Alg maintained high cell viability for up to 10 days (Supplementary Fig. S7). Additionally, cells in both 1% Alg and 2% Alg proliferated and increased in number starting from day 3, which became statistically significant on days 7 and 10 (Fig. 3A). Remarkably, a higher T cell proliferation was observed in 1% Alg, where the total cell number was significantly higher than in 2% Alg at all time points from day 3 onwards (Fig. 3A). Additionally, maximum projection of 3D confocal images also showed this trend, where differences in cell morphology and organization between the two hydrogels were evident (Fig. 3B). While compact and spherical cell clusters were observed in 2% Alg, spread cell aggregates with larger and more dispersed structures were found in 1% Alg, as revealed by DAPI/PHA staining (Fig. 3B).

#### 3.4.2 T cell release from click alginate hydrogels

To analyze the release of cells from these hydrogels, 50 μL of the hydrogels were injected into a transwell and cultured for 10 days. On days 1, 3, 7, and 10, the cells outside the gel present in the culture media were counted. For visual evaluation of the release profiles, Jurkat-TOM cells were encapsulated in non-injected cylindrical gels and cultured without a transwell system in a well plate. On equivalent time points, Jurkat-TOM release was monitored by fluorescence imaging.

Both hydrogels demonstrated the ability to release live cells into the medium, as cells outside the hydrogels were counted and visualized in both 1% Alg and 2% Alg (Fig. 3C and D). On day 3, an increase in released cells was observed, especially in 1% Alg (Fig. 3C). From day 7 onwards, significantly more released cells were found in both 1% Alg and 2% Alg compared to day 0. When comparing 1% Alg and 2% Alg, cell release was significantly higher in 1% Alg from day 7 onwards (Fig. 3C). Fluorescence images confirmed this, showing a higher number of Jurkat-TOM cells out of the gels (marked by the white dashed line) over time, particularly in 1% Alg (Fig. 3D).

#### 3.4.3 T cell functional phenotype based on cytokine secretion analysis

To evaluate the functional integrity of T cells following encapsulation, cytokine release was monitored over a 10-day period. Specifically, IL-2 and IFN-ℽ secretion levels in 1% Alg were quantified and compared against a standard 2D culture control.

The analysis revealed no significant differences in cytokine secretion between encapsulated T cells and those in 2D cultures, indicating that T cell functionality is fully preserved after encapsulation in 1% Alg (Supplementary Fig. S8). Furthermore, this functional state remained stable over time, as evidenced by the lack of significant differences in cytokine production between day 1 and day 10 (Supplementary Fig. S8).

### 3.5 Click alginate hydrogels improve T cell administration and persistence *in vivo*

#### 3.5.1 T cell administration and local persistence using click alginate hydrogels in an *in vivo* CAM model

In the *in vivo CAM* model, T cell delivery and spatio-temporal persistence were evaluated using longitudinal BLI analysis with an IVIS. Jurkat-TOM cells were encapsulated in 1% Alg and 2% Alg, with a bolus injection in culture medium serving as a control, and were injected in the proximal CAM.

**Fig. 4:**
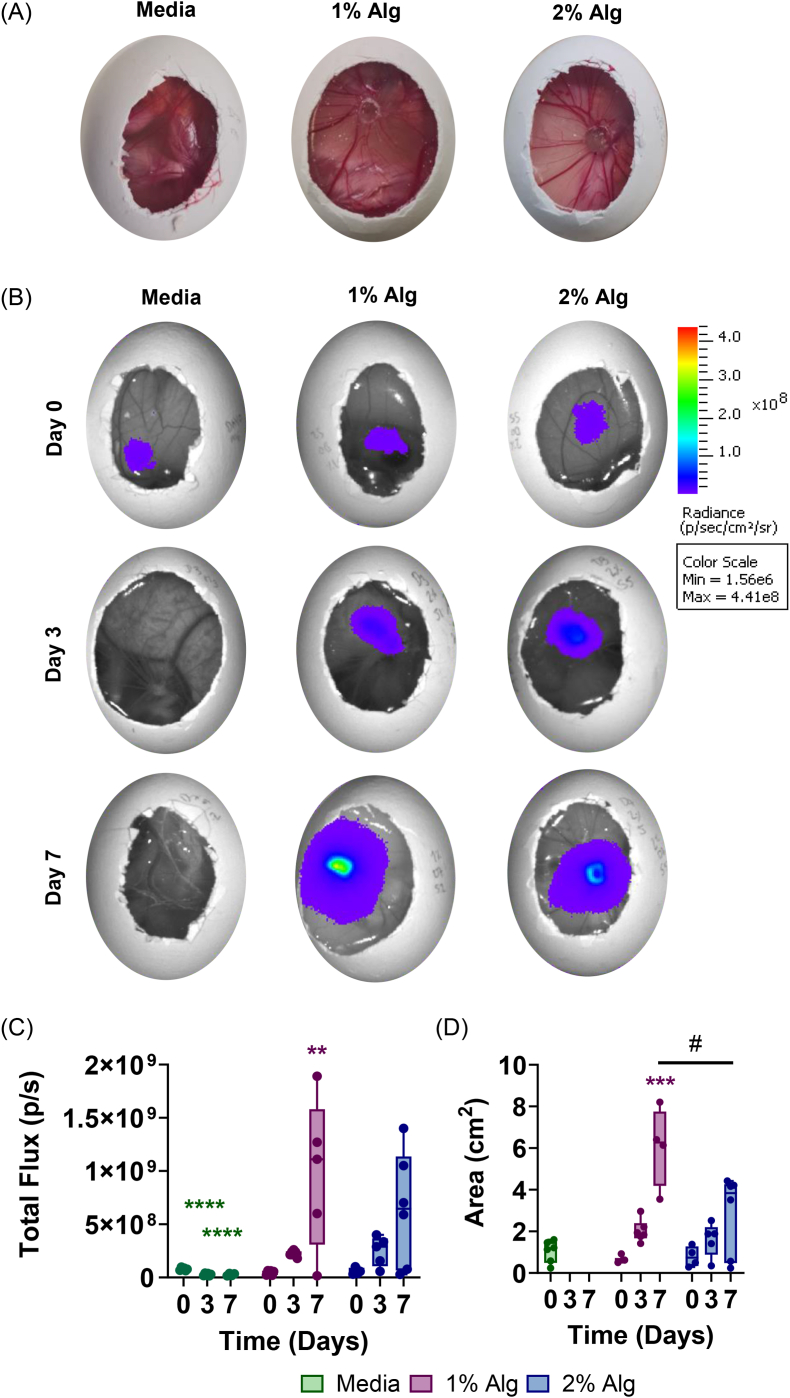
*In vivo* evaluation of T cell administration and persistence in the CAM model. Representative images of the proximal CAM at the end of the experiment at day 7 (A), IVIS images of eggs at different time points (B), quantification of the BLI signal in total flux (p/s) (C) and BLI signal area (D). Box plots show the median, 1^st^ and 3^rd^ quartiles, and min and max values; n = 4-5 per group. Comparisons between 1% Alg and 2% Alg were performed using a Mann-Whitney test. Statistically significant differences are represented with #. Comparisons within each group, with respect to time 0 were performed by Friedman's test. Statistically significant differences are represented with ∗. (For interpretation of the references to color in this figure legend, the reader is referred to the Web version of this article).

The chick embryos were able to continue their development upon hydrogel injection. Before sacrifice, the proximal CAM, where the injection was performed, showed blood vessels around both 1% Alg and 2% Alg hydrogels, indicating biocompatibility and a correct tissue integration (Fig. 4A). The BLI results showed that both 1% Alg and 2% Alg supported cell viability and proliferation *in vivo,* as shown by the increase in total flux with respect to day 0 (Fig. 4B and C). However, the increase on day 7 with respect to day 0 was only statistically significant in 1% Alg, but not in 2% Alg (Fig. 4 C). Notably, from day 3 onwards, no BLI signal was detected in the eggs that received the bolus injection (Fig. 4B, Supplementary Fig. S9). When analyzing the signal area, 1% Alg presented a significantly larger area than 2% Alg (Fig. 4D). Considering these results along with the *in vitro* results, 1% Alg was chosen for the *in vivo* mouse model.

#### 3.5.2 T cell administration and local persistence using click alginate hydrogels in a mouse model

To assess the transplantation efficacy and spatio-temporal persistence of T cells in a mouse model, Jurkat-TOM were encapsulated in 1% Alg or suspended in culture medium (as a bolus control) and injected in the fourth mammary gland. T cell persistence in the target organ was evaluated by *in vivo* and *ex vivo* IVIS imaging. Histology and immunofluorescence were performed to assess hydrogel integration and T cell detection in the injected organ.

**Fig. 5:**
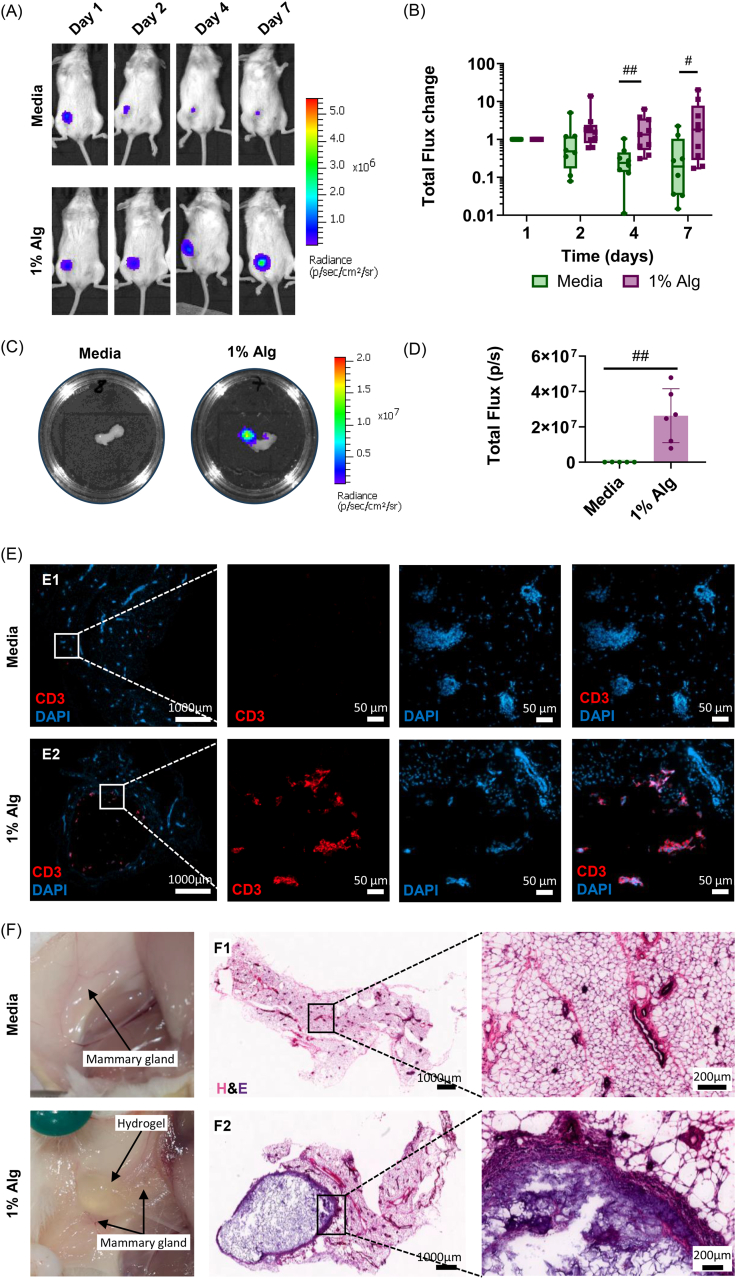
*In vivo* and *ex vivo* evaluation of T cell administration and persistence in the mouse model. Representative *in vivo* BLI images at different time points after injection (A), quantification of the *in vivo* BLI signal change with respect to day 1 (Total flux/Total flux on day 1) of 1% Alg in magenta and bolus injection with media in green (B), representative *ex vivo* BLI images of mammary gland explants (C), quantification of the *ex vivo* BLI signal of 1% Alg in magenta and media in green (D), detection of injected T cells in the mammary gland by CD3^+^ immunofluorescence (E), where E1 and E2 show the entire mammary tissues and the following three are the corresponding magnified views, and representative H&E staining of injected mammary glands (F), where the left images are photographs of the injected mammary glands, F1 and F2 show the H&E staining of the whole mammary tissues and each is followed by a corresponding magnified view. For B, box plots show the median, 1^st^ and 3^rd^ quartiles, and min and max values; n = 8-9 per group. For D, bar plots represent mean ± standard deviation, n = 6 per group. Comparisons between 1% Alg and media were performed using a Mann-Whitney test in B and a *t*-test in D. Statistically significant differences between groups are represented with #. (For interpretation of the references to color in this figure legend, the reader is referred to the Web version of this article).

On day 1, the BLI signal was similar in medium and 1% Alg; however, from day 4 onwards a significantly lower signal was found in mice that received cells in culture medium compared to those in which 1% Alg was used (Fig. 5A and B). On day 7, the signal intensity in the 1% Alg group remained detectable and localized to the injection site, whereas in the bolus injection group the signal decreased and was barely detectable in most mice (Supplementary Fig. S10). *Ex vivo* IVIS imaging of the mammary gland explants after sacrifice confirmed these results, as BLI signal was only detectable in mammary glands where 1% Alg was injected (Fig. 5C and D, Supplementary Fig. S11).

Further confirmation of T cells in the mammary glands was obtained using CD3^+^ immunofluorescence staining (Fig. 5E). Injected T cells were only detected in mammary glands in which 1% Alg was used as the injection method (Fig. 5E2), whereas T cells were absent in mice that received T cells in media only (Fig. 5E1).

Histological analysis of the injected mammary glands with H&E staining (Fig. 5F) also showed that 1% Alg successfully integrated into the mammary gland tissue, and cell and tissue infiltration through the hydrogel was observed (Fig. 5F2).

## 4 Discussion

In this study, we developed injectable and viscoelastic click alginate hydrogels, using LMW oxidized alginate and N:T click crosslinking by the spontaneous IEDDA reaction, to improve the local delivery and efficacy of T cell therapies compared to bolus administration. This platform addresses the limitations of many current biomaterials, which often rely on rigid structures, synthetic nature, or external triggers like UV light and photoinitiators that hinder clinical translation [2,20,21]. By modifying the alginate polymer concentration and the ratio of N:T crosslinks, we generated a soft, viscoelastic and degradable matrix that remains injectable even after full crosslinking, offering biocompatible and clinically relevant characteristics [38,57]. These properties preserve T cell viability and phenotype, while enhancing their proliferation and improving spatio-temporal administration *in vivo*. This work deviates from designs focused on rigid materials and opens a new way toward clinically injectable devices for T cell administration, proposing soft but stable injectable hydrogels.

Recent research defines an “easy to inject” hydrogel as one requiring a manual force below 12 N (using a 5 mL syringe with a 19 G or 31 G needle at 0.2 and 1 mm/s) [58]. In this work, the injectability analysis showed that 1% Alg required significantly lower injection forces than 2% Alg, consistently falling below the clinical threshold of 12 N. This implies that 1% Alg can be easily administered by all clinicians, regardless of age or gender [58]. While injectability in covalent networks is often achieved via dynamic bonds or *in situ* gelation [25,27,59,60], our results suggest that the softer, less crosslinked structure of 1% Alg facilitates flow under shear conditions even in a fully crosslinked state, reducing resistance. The shear forces are particularly relevant, as they can cause cell damage during needle extrusion, compromising cell viability, which is key for the therapeutic efficiency after administration [[61], [62], [63]]. Additionally, the lower alginate concentration, and consequent reduction in crosslinking reactions, minimized N_2_ gas generation during the process, maintaining high cell viability after injection [64]. This is reflected in the analysis of the cell viability after injection, which remained above 75% in 1% Alg, while it decreased to 63% with 2% Alg. Crucially, non-injected cylindrical hydrogels showed high cell viability throughout the 10-day study, confirming that the hydrogels themselves are cytocompatible and that the observed cell death after injection is primarily attributable to shear stress rather than material toxicity.

Rheological characterization confirmed that polymer concentration significantly influences crosslinking kinetics and viscoelastic properties. The lower alginate concentration of 1% Alg resulted in a softer, more porous, and permeable structure, increasing nutrient diffusion, cell proliferation and migration compared to 2% Alg [65,66]. Some 2D studies often show that stiffer substrates promote T cell proliferation and IL-2 secretion [67,68]. However, these findings differ from 3D environments [69]. A study using nanotextured elastic platforms showed that T cells primarily move through amoeboid migration, characterized by a rounded morphology [70,71]. Weiden et al. used polyisocyanopeptide hydrogels (defining "soft" as 30 Pa and "rigid" as 400 Pa) and showed that the migration capacity of T cells actually decreases with increasing stiffness due to smaller pore sizes and physical constraints [72]. These results are consistent with our observations, suggesting that the smaller mesh size and higher stiffness of 2% Alg could hinder T cell release, and instead induce the formation of cell clusters confined within the hydrogel. Furthermore, in 1% Alg, cells were observed outside the hydrogel as early as day 1 (Fig. 3), even before significant degradation of the matrix (Suppl. Fig. S5). This suggests that T cell release from 1% Alg results from an interplay between active migration facilitated by rheological properties and porosity of the hydrogel, further enhanced by hydrogel degradation [10].

Viscoelasticity of the matrix regulates not only the behavior of individual cells but also their collective behavior, reflecting how cells interact with the ECM through dynamic processes that span a range of timescales, from milliseconds to hours [26,39,73,74]**.** 1% Alg showed a viscous relaxation within 10^3^- 10^4^ s, a timescale significantly larger than ionically crosslinked alginates (τ_1/2_ =10 - 10^1^ s) and most native soft tissues [26,75]. Although stiffness and viscoelasticity were not decoupled in this study, reported stress relaxation rates within our range have been shown to exert distinct effects across various cell types, suggesting they may also influence T cell responses. For instance, τ_1/2_ similar to ours described by Roth et al. showed that “fast-relaxing" matrices (τ_1/2_ = 10^4^ s) enhance neurite outgrowth and differentiation compared to slower relaxing systems (τ_1/2_ = 10^5^ s) [42]. Additionally, Morgan et al. observed that relaxation times between 10^4^ and 10^5^ s dictate cytoskeletal organization [73]. Furthermore, in immune cells, slow-relaxing matrices (τ_1/2_ =10^3^ s) favor proinflammatory monocyte polarization, whereas faster relaxation preserves immature phenotypes [76].

Regarding T cell responses specifically, both matrix stiffness and viscoelasticity have been shown to play a critical role in their activation, function and differentiation [[47], [48], [49]]. Recently, Jeffreys et al. demonstrated that combining stiffness and stress relaxation has a striking effect on lineage biasing. Their study showed that both soft-viscous (G' = 400 Pa, τ_1/2_ = 10 s) and soft-elastic (G' = 400 Pa, τ_1/2_ = 100 s) matrices significantly drive T cell lineage commitment by day 7. In contrast, stiff-elastic matrices (G' = 1000 Pa, τ_1/2_ = 100 s) support the myeloid lineage, highlighting the synergy between these mechanical cues in T cell differentiation. Another study comparing elastic with viscoelastic microspheres demonstrated that the latter lead to more effective T cell activation [47]. Furthermore, it has been reported that ECM viscoelasticity modulates the transcriptional landscape of T cells, with slow-relaxing matrices (τ_1/2_ = 10^3^ - 10^4^ s) enhancing activation markers and fast-relaxing ones (τ_1/2_ = 1 - 10 s) promoting memory markers. The highest proliferation observed in 1% Alg suggests that its specific stress relaxation profile could facilitate superior T cell activation, promoting expansion and migration while sustaining functional integrity [77]. Taken together, these findings highlight the importance of incorporating viscoelastic properties into the matrix to drive T cell proliferation and release.

Following established protocols by Adu-Berchie et al. [78], our 7-day monitoring period was sufficient to demonstrate an improved local T cell retention and persistence using 1% Alg hydrogel compared to conventional bolus injections, while adhering to ethical animal welfare “Refinement” principles. Comparing the presented hydrogel to some implantable systems, alginate matrices have been combined with functionalized silica microparticles to deliver T cells for 12 days [79], and patient-derived lyophilized lymph nodes have enabled the sustained release for up to 24 days [77]. While effective, these platforms require surgical placement. In contrast, 1% Alg hydrogel offers a less invasive alternative, as it is injectable via a syringe. Alternatively, *in situ* forming chitosan hydrogels allow delivery over 14 days [80], but can present challenges regarding gelation kinetics *in vivo*. Our pre-formed yet injectable 1% Alg provides a more robust and predictable material. Moving towards more complex architectures, the synergistic *in situ* vaccination-enhanced T cell depot (SIVET) locally delivers T cells while recruiting host cells [78]. While SIVET is a sophisticated multifunctional system, it relies on macroporosity and multiple recruitment factors for T cell motility. Similarly, the mechanical contribution of viscoelasticity to T cell movement within these macroporous hyaluronic acid (HA) scaffolds remains poorly defined; consequently, T cell migration is restricted to fixed pore architectures. In contrast, 1% Alg achieves T cell movement through the soft matrix, with an open mesh structure and its inherent viscoelasticity. Furthermore, while microneedle patches are useful for superficial applications [81], an injectable hydrogel offers greater versatility for reaching internal sites, as it can be easily administered into hard-to-access areas. As a next step, validation of this hydrogel for spatio-temporal T cell immunotherapy using isolated primary T cells is essential to evaluate its efficacy in more complex physiological environments.

In conclusion, here we developed injectable and viscoelastic click alginate hydrogels, using LMW oxidized alginate and spontaneous N:T click crosslinking. 1% Alg hydrogels are soft, viscoelastic, degradable, and importantly, enable injectability even after full crosslinking, without compromising the viability of encapsulated T cells. We show that 1% Alg hydrogel acts as a T cell reservoir to enhance the administration, survival, proliferation, local persistence and prolonged release *in vitro* and *in vivo*. By improving the local delivery and efficacy of T cell therapies compared to bolus administration, this injectable hydrogel could have future applications for local *in vivo* administration of T cell immunotherapies.

## Funding sources

J.B, U.H and A.R.V would like to acknowledge funding from Hezkuntza Saila Eusko Jaurlaritza (PRE_2024_2_0062, PRE_2024_2_0005 and PRE_2024_2_0009 respectively). S.M. is funded by a Sara Borrell Fellowship from Instituto de Salud Carlos III (ISCIII) (CD23/00059), co-funded by the European Union. O.M.I. was recipient of a Postdoctoral grant from the Fundación Científica Asociación Española Contra el Cáncer (POSTD234723MITX). M.M.C would like to thank funding from the 10.13039/501100004837Spanish Ministry of Science and Innovation (CNS2023-145020) and the 10.13039/501100004587ISCIII (PI21/01208), co-funded by the 10.13039/501100000780European Union. R.A would like to acknowledge funding from the María de Maeztu Excellence Unit CEX2023-001303-M funded by 10.13039/501100004837MCIN/10.13039/501100011033AEI/10.13039/501100011033, from Hezkuntza Saila 10.13039/501100003086Eusko Jaurlaritza (grant IT1503-22) and from Osasun Saila 10.13039/501100003086Eusko Jaurlaritza (grant 2024333015). A.C., A.P, and M.M.C. would like to acknowledge funding from Osasun Saila 10.13039/501100003086Eusko Jaurlaritza (grant CART-gel 2022333036; CART-gel - II, 2023333027; CART-gel - III, 2024333001) and from IKERBASQUE Basque Foundation for Science. A.C. would like to thank funding from Fundación Científica Asociación Española Contra el Cáncer (grant LABAE223466CIPI), from the 10.13039/501100004837Spanish Ministry of Science and Innovation (10.13039/501100004837MCIN/10.13039/501100011033AEI/10.13039/501100011033/10.13039/501100002924FEDER 10.13039/501100012637UE, grant PID2021-123013OB-I00) and from the 10.13039/501100000781European Research Council Consolidator Grant (DORMATRIX, 101123883).

## CRediT authorship contribution statement

**Jone Berasain:** Conceptualization, Data curation, Formal analysis, Funding acquisition, Investigation, Methodology, Software, Validation, Visualization, Writing – original draft, Writing – review & editing. **Sara Manzano:** Investigation, Methodology, Writing – review & editing. **Oihane Mitxelena-Iribarren:** Data curation, Software, Writing – review & editing. **Unai Heras:** Data curation, Software, Writing – review & editing. **Ainhoa Romo-Valera:** Investigation, Writing – review & editing. **Peio Azcoaga:** Investigation, Methodology, Writing – review & editing. **Leire Iturriaga:** Investigation, Writing – review & editing. **Leire Egia-Mendikute:** Methodology, Validation, Writing – review & editing. **Asís Palazón:** Funding acquisition, Resources, Validation, Writing – review & editing. **Mercedes Fernández:** Methodology, Writing – review & editing. **María M. Caffarel:** Funding acquisition, Methodology, Resources, Validation, Writing – review & editing. **Robert Aguirresarobe:** Methodology, Project administration, Resources, Supervision, Validation, Writing – review & editing. **Amaia Cipitria:** Conceptualization, Funding acquisition, Methodology, Project administration, Resources, Supervision, Validation, Writing – original draft, Writing – review & editing.

## Declaration of competing interest

The authors declare that they have no known competing financial interests or personal relationships that could have appeared to influence the work reported in this paper.

## Data Availability

All raw and processed data and the Dragonfly models used are available in a publicly accessible Zenodo repository: .https://doi.org/10.5281/zenodo.16961174.
