# Supplementary material for: Injectable and viscoelastic click alginate hydrogels for spatio-temporal T cell administration in vivo
Source: Mater Today Bio. 2026 Apr 24;38:103128. doi: 10.1016/j.mtbio.2026.103128 (PMC13156761; doi:10.1016/j.mtbio.2026.103128)
Supplement: Multimedia component 1 [file mmc1.pdf]

Supplementary Material

Injectable and viscoelastic click alginate hydrogels for spatio-temporal T cell administration in vivo

Jone Berasain, Sara Manzano, Oihane Mitxelena-Iribarren, Unai Heras, Ainhoa Romo-Valera, Peio Azcoaga, Leire Iturriaga, Leire Egia-Mendikute, Asís Palazón, Mercedes Fernández, María M Caffarel, Robert Aguirresarobe, Amaia Cipitria

- Supplementary Figure S1. ^{1}H NMR spectra of oxidized and modified LMW alginate with norbornene (N) and tetrazine (T).
- Supplementary Table S1. DS_{actual} of N-mod and T-mod from two independent synthesis batches in oxidized LMW alginate.
- Supplementary Figure S2. Oxidized and functionalized alginate with norbornene and tetrazine for covalent crosslinking by IEDDA click reaction.
- Supplementary Figure S3. Strain sweep test. G' and G'' of 1% Alg in magenta and 2% Alg in blue.
- Supplementary Figure S4. Morphological characterization of lyophilized hydrogels with 3D µCT imaging.
- Supplementary Figure S5. Degradability assay based on wet weight and dry weight changes over time.
- Supplementary Figure S6. Injectability test at different time points (0 min to 180 min) during the gelation process.
- Supplementary Figure S7. T cell encapsulation in non-injected cylindrical hydrogels and cell viability.
- Supplementary Figure S8. Cytokine secretion analysis of encapsulated T cells.
- Supplementary Figure S9. BLI signal in the in vivo CAM model comparing bolus injection in media, 1% Alg and 2% Alg, at day 0, 3 and 7.
- Supplementary Figure S10. In vivo BLI signal in mouse model comparing bolus injection in media vs. 1% Alg, at days 1, 2, 4 and 7.
- Supplementary Figure S11. Supplementary Figure S12. Ex vivo BLI imaging of the injected mammary glands comparing bolus injection in media vs. 1% Alg.

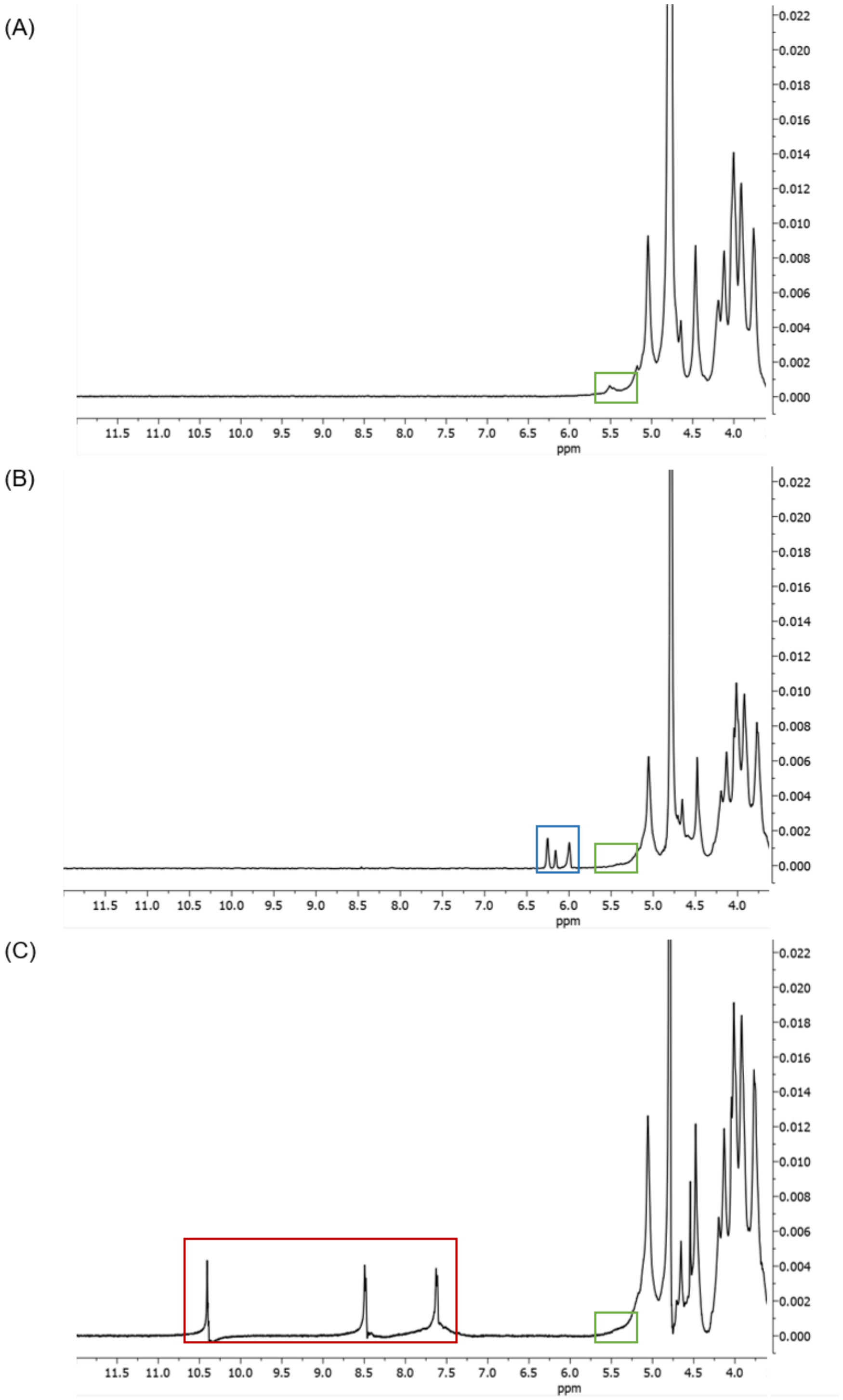

Supplementary Figure S1. ^{1}H NMR spectra of oxidized and modified LMW alginate with norbornene (N) and tetrazine (T). Alginate polymer after oxidation (A), reduction and modification with N (B), and reduction and modification with T (C), demonstrating the disappearance of the aldehyde peak at 5.5 ppm (highlighted by the green box), appearance of automatically detected N peaks between 6.5 - 6 ppm (blue box) and T peaks at 10.5, 8.5 and 7.5 ppm (red box).

Synthesis batch	N-mod	T-mod
1st	18.71	27.72
2nd	21.26	29.91

Supplementary Table S1. DS_{actual} of N-mod and T-mod from two independent synthesis batches in oxidized LMW alginate.

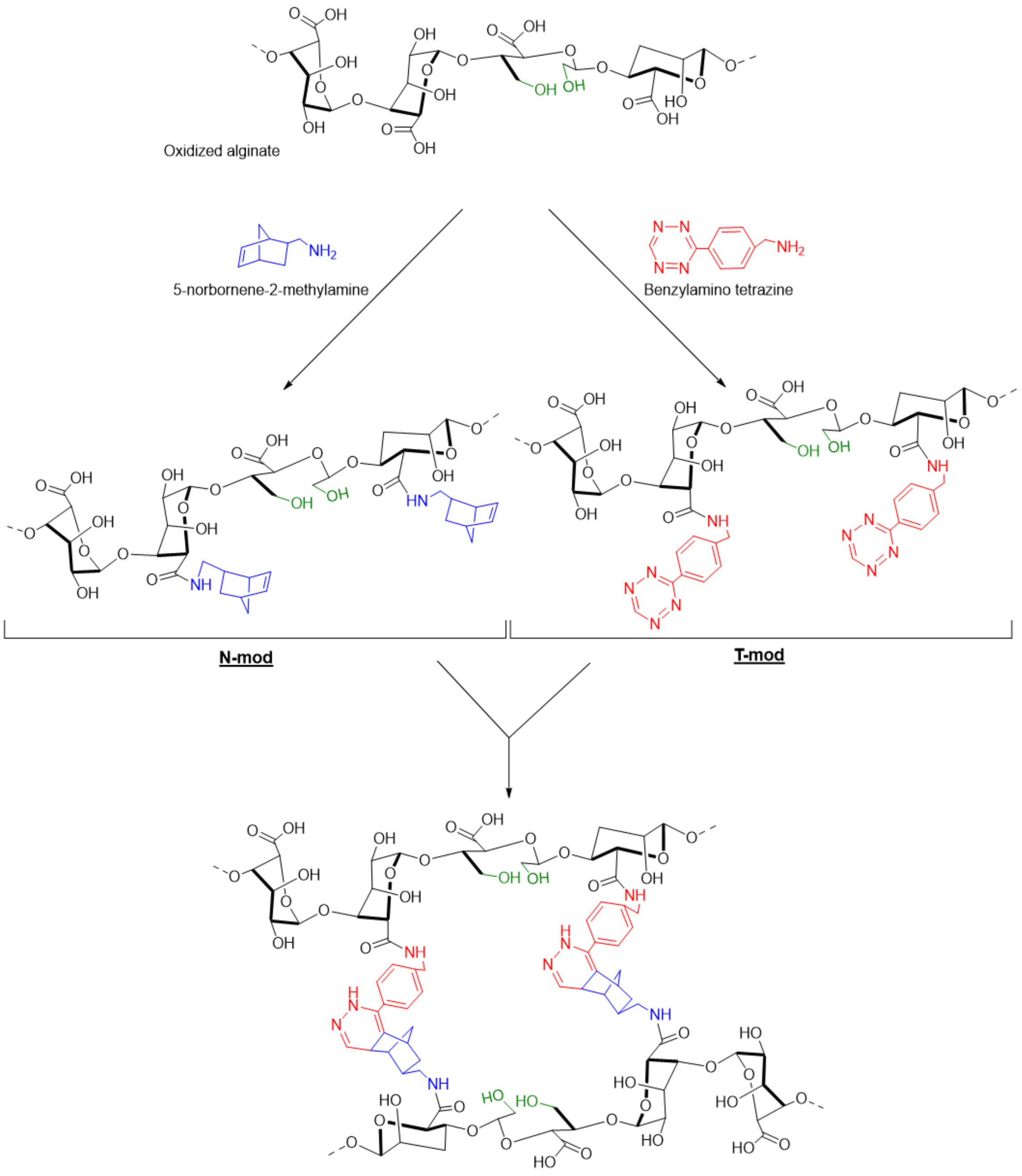

Supplementary Figure S2. Oxidized and functionalized alginate with norbornene and tetrazine for covalent crosslinking by IEDDA click reaction. Open chains susceptible to hydrolysis in green, N-mod (blue) and T-mod (red) generation, and click crosslinking between N:T.

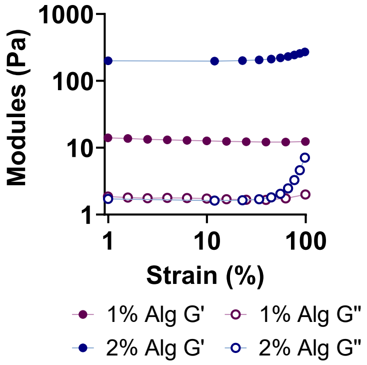

Supplementary Figure S3. Strain sweep test. G’ and G’’ of 1% Alg in magenta and 2% Alg in blue.

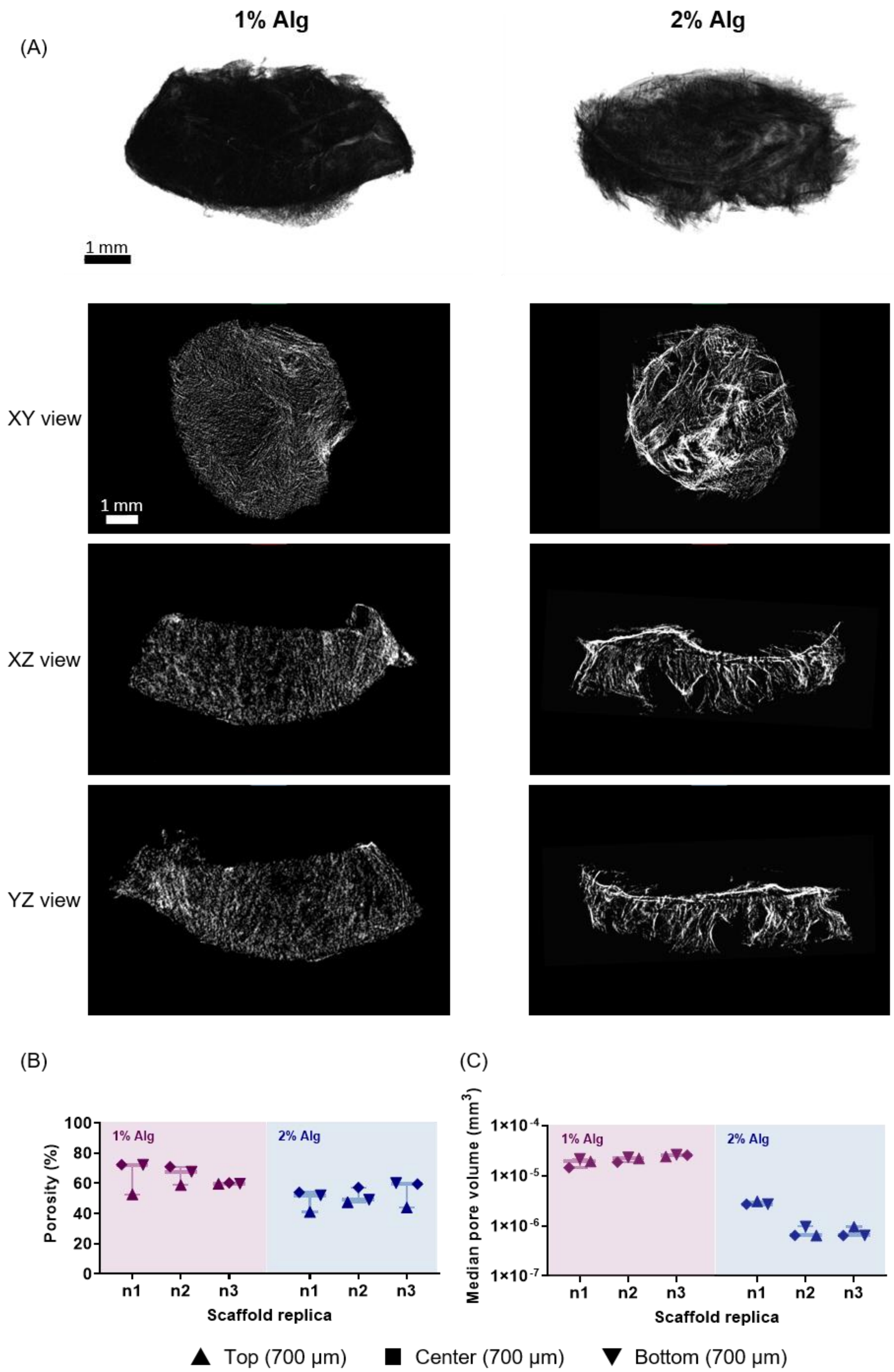

Supplementary Figure S4. Morphological characterization of lyophilized hydrogels with 3D µCT imaging. 3D reconstruction and orthogonal views (XY, XZ, YZ planes) obtained via micro-CT of 1% Alg (left) and 2% Alg (right) hydrogels (A). Quantification of porosity (B) and pore size distribution (C) across the hydrogel height divided in subsections of 700 µm in the top, center and bottom.

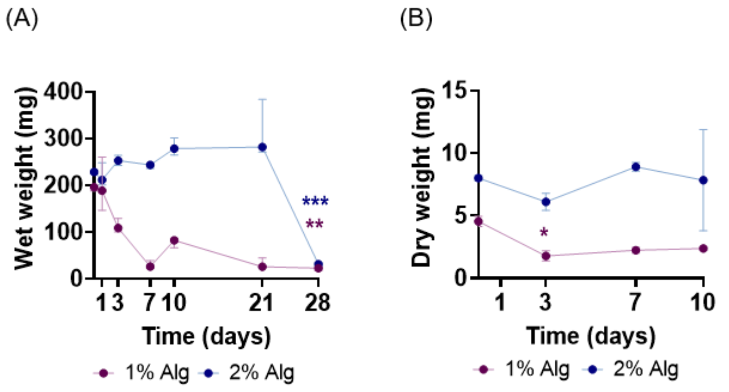

Supplementary Figure S5. Degradability assay based on wet weight and dry weight changes over time. Wet weight changes in RPMI medium at 37 °C under a CO_2 atmosphere (A) and dry weight changes measured after lyophilization (B) of 1% Alg in magenta and 2% Alg in blue of n = 3 per group. Median +/- standard deviation are represented. Comparisons within each group, with respect to time 0 was performed by Mann Whitney. Statistically significant differences are represented with *.

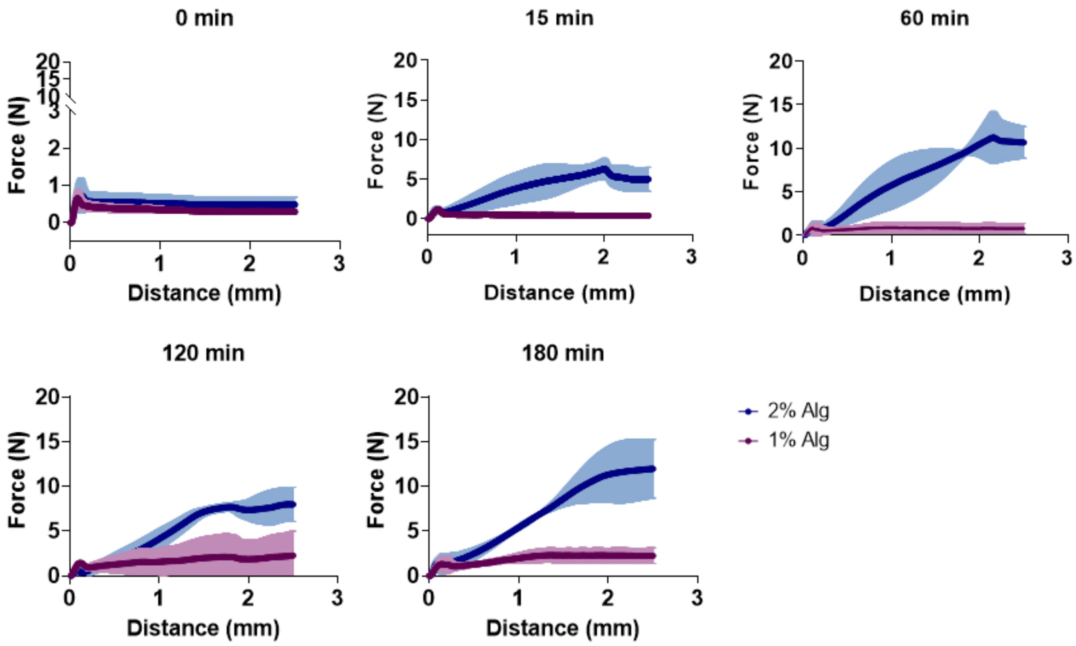

Supplementary Figure S6. Injectability test at different time points (0 min to 180 min) during the gelation process. Injection force profiles of 1% Alg in magenta and 2% Alg in blue. Mean +/- standard deviation of n = 3 per group are represented.

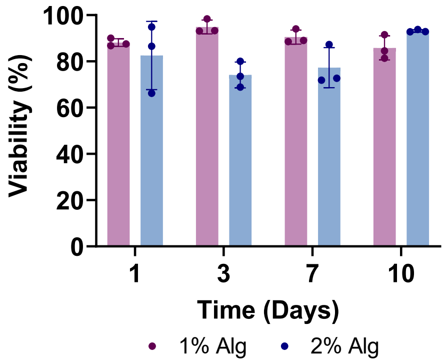

Supplementary Figure S7. T cell encapsulation in non-injected cylindrical hydrogels and cell viability. Percentage of live T cells encapsulated in 1% Alg in magenta and 2% Alg in blue.

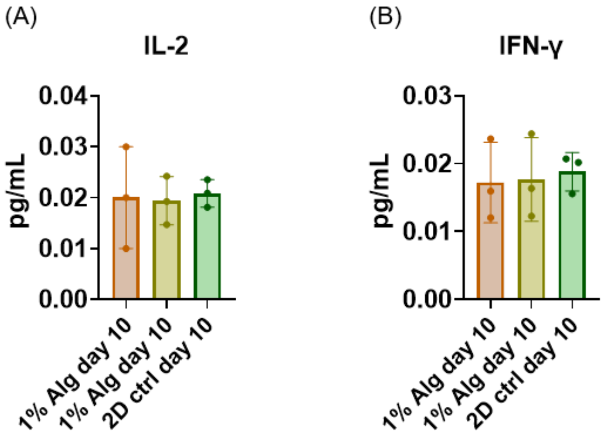

Supplementary Figure S8. Cytokine secretion analysis of encapsulated T cells. IL-2 (A) and IFN-γ (B) secretion of encapsulated T cells in 1% Alg at day 0 and day 10, compared to a 2D control. Bar plots represent mean +/- standard deviation, n = 3 per group. Comparisons between groups were performed by one-way ANOVA. Statistically significant differences are represented with *.

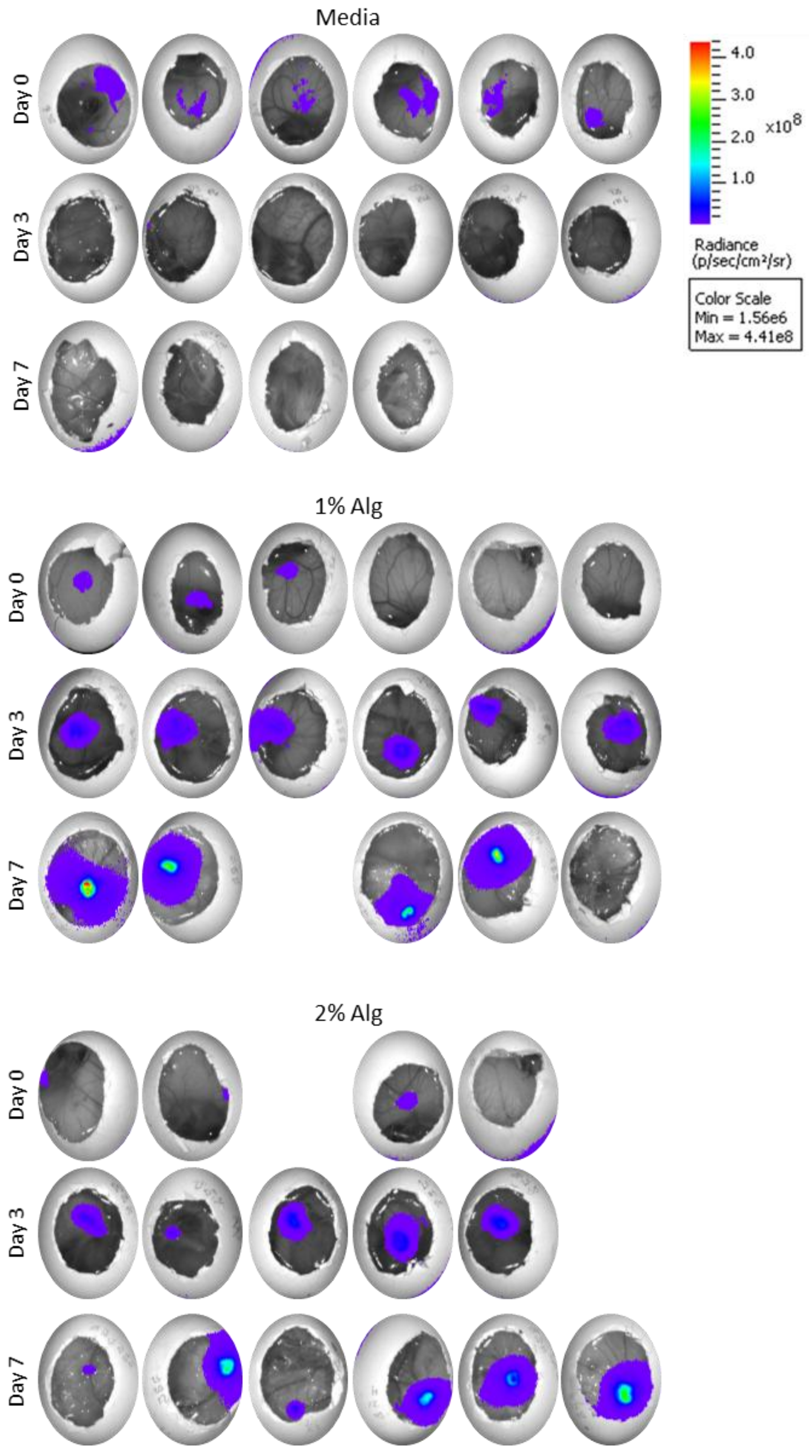

Supplementary Figure S9. BLI signal in the in vivo CAM model comparing bolus injection in media, 1% Alg and 2% Alg, at day 0, 3 and 7.

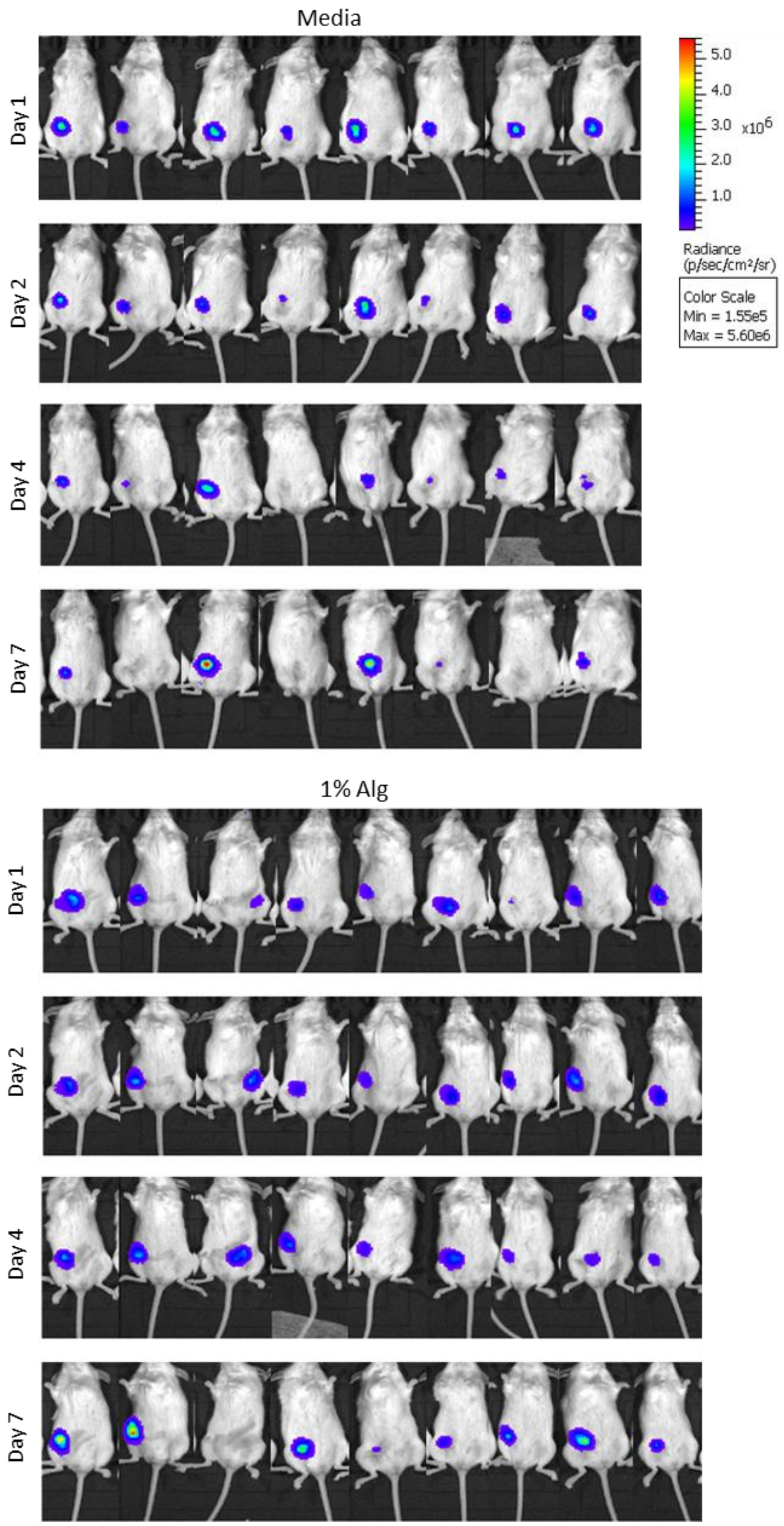

Supplementary Figure S10. In vivo BLI signal in mouse model comparing bolus injection in media vs. 1% Alg, at days 1, 2, 4 and 7.

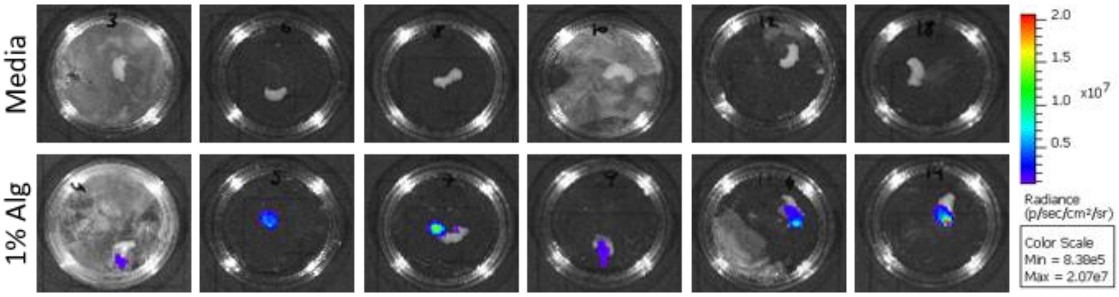

Supplementary Figure S11. Ex vivo BLI imaging of the injected mammary glands comparing bolus injection in media vs. 1% Alg.
